# Supplementary material for: In vitro high-content screening reveals miR-429 as a protective molecule in photoreceptor degeneration
Source: Mol Ther Nucleic Acids. 2024 Dec 22;36(1):102434. doi: 10.1016/j.omtn.2024.102434 (PMC11773019; doi:10.1016/j.omtn.2024.102434)
Supplement: Document S1. Figures S1–S4 and Table S2 [file mmc1.pdf]

OMTN, Volume *36*

Supplemental information

In vitro high-content screening reveals miR-429 as a protective molecule in photoreceptor degeneration

Georgios Petrogiannakis, Irene Guadagnino, Santiago Negueruela, Martina Di Guida, Elena Marrocco, Mariateresa Pizzo, Annalaura Torella, Mariateresa Zanobio, Marianthi Karali, Diego Luis Medina, Sabrina Carrella, and Sandro Banfi

Table S1: HCI results of all tested miRNAs

Table S2: Gene Ontology biological processes enriched in response to miR-429 overexpression

pathway	pval	padj	log2 err	ES	NES	size
GOBP_INTERLEUKIN_10_PRODUCTION	2.57E-07	1.20E-04	0.67	-0.69	-2.21	53
GOBP_REGULATION_OF_LYMPHOCYTE_MEDIATED_IMMUNITY	9.09E-07	2.06E-04	0.66	-0.51	-1.90	157
GOBP_B_CELL_MEDIATED_IMMUNITY	3.61E-06	4.40E-04	0.63	-0.51	-1.88	143
GOBP_REGULATION_OF_PHAGOCYTOSIS	4.43E-06	5.10E-04	0.61	-0.58	-2.01	86
GOBP_NEGATIVE_REGULATION_OF_LEUKOCYTE_MEDIATED_IMMUNITY	1.69E-05	1.60E-03	0.58	-0.63	-2.09	66
GOBP_NEGATIVE_REGULATION_OF_LYMPHOCYTE_MEDIATED_IMMUNITY	2.01E-05	1.86E-03	0.58	-0.63	-2.02	54
GOBP_CELLULAR_RESPONSE_TO_AMYLOID_BETA	2.48E-05	2.03E-03	0.58	-0.68	-2.05	40
GOBP_NEGATIVE_REGULATION_OF_IMMUNE_EFFECTOR_PROCESS	2.59E-05	2.04E-03	0.58	-0.52	-1.86	107
GOBP_RESPONSE_TO_AMYLOID_BETA	2.72E-05	2.10E-03	0.58	-0.66	-2.06	49
GOBP_HUMORAL_IMMUNE_RESPONSE_MEDIATED_BY_CIRCULATING_IMMUNOGLOBULIN	2.97E-05	2.18E-03	0.58	-0.61	-2.01	64
GOBP_MEMBRANE_INVAGINATION	3.48E-05	2.51E-03	0.56	-0.53	-1.84	87
GOBP_REGULATION_OF_T_CELL_MEDIATED_IMMUNITY	3.84E-05	2.71E-03	0.56	-0.56	-1.92	79
GOBP_MACROPHAGE_ACTIVATION	4.31E-05	2.87E-03	0.56	-0.54	-1.86	95
GOBP_POSITIVE_REGULATION_OF_INTERLEUKIN_10_PRODUCTION	4.59E-05	2.91E-03	0.56	-0.68	-2.00	38
GOBP_POSITIVE_REGULATION_OF_PHAGOCYTOSIS	4.72E-05	2.91E-03	0.56	-0.62	-2.03	58
GOBP_NEGATIVE_REGULATION_OF_CELL_KILLING	5.13E-05	3.12E-03	0.56	-0.71	-1.98	30
GOBP_INTERFERON_GAMMA_PRODUCTION	7.57E-05	4.08E-03	0.54	-0.52	-1.82	101
GOBP_REGULATION_OF_CELL_KILLING	8.90E-05	4.68E-03	0.54	-0.53	-1.85	93
GOBP_CELLULAR_EXTRAVASATION	1.00E-04	5.07E-03	0.54	-0.56	-1.87	67
GOBP_COMPLEMENT_ACTIVATION	1.12E-04	5.47E-03	0.54	-0.56	-1.88	69
GOBP_DETECTION_OF_LIGHT_STIMULUS	1.29E-04	6.23E-03	0.52	0.59	1.86	58
GOBP_LENS_DEVELOPMENT_IN_CAMERA_TYPE_EYE	1.54E-04	7.20E-03	0.52	0.54	1.83	80
GOBP_PHOTORECEPTOR_CELL_DEVELOPMENT	2.25E-04	9.20E-03	0.52	0.61	1.88	48
GOBP_POSITIVE_REGULATION_OF_INTERFERON_GAMMA_PRODUCTION	2.76E-04	1.10E-02	0.50	-0.54	-1.82	71
GOBP_EYE_PHOTORECEPTOR_CELL_DEVELOPMENT	3.05E-04	1.18E-02	0.50	0.64	1.86	34
GOBP_REGULATION_OF_T_CELL_MEDIATED_CYTOTOXICITY	3.25E-04	1.25E-02	0.50	-0.65	-1.87	33
GOBP_DETECTION_OF_VISIBLE_LIGHT	4.24E-04	1.56E-02	0.50	0.61	1.87	45
GOBP_POSITIVE_REGULATION_OF_TYROSINE_PHOSPHORYLATION_OF_STAT_PROTEIN	4.38E-04	1.57E-02	0.50	-0.57	-1.85	56
GOBP_TYROSINE_PHOSPHORYLATION_OF_STAT_PROTEIN	5.23E-04	1.77E-02	0.48	-0.54	-1.78	69
GOBP_REGULATION_OF_LEUKOCYTE_MEDIATED_CYTOTOXICITY	5.43E-04	1.82E-02	0.48	-0.52	-1.76	74
GOBP_POSITIVE_REGULATION_OF_CALCIUM_MEDIATED_SIGNALING	5.76E-04	1.89E-02	0.48	-0.67	-1.90	32
GOBP_REGULATION_OF_B_CELL_MEDIATED_IMMUNITY	6.75E-04	2.14E-02	0.48	-0.55	-1.76	55
GOBP_B_CELL_PROLIFERATION	6.97E-04	2.18E-02	0.48	-0.52	-1.78	82
GOBP_FC_GAMMA_RECEPTOR_SIGNALING_PATHWAY	7.41E-04	2.29E-02	0.48	-0.65	-1.81	30
GOBP_EYE_PHOTORECEPTOR_CELL_DIFFERENTIATION	7.62E-04	2.30E-02	0.48	0.57	1.74	44
GOBP_INTERLEUKIN_12_PRODUCTION	7.62E-04	2.30E-02	0.48	-0.54	-1.78	63
GOBP_NEUROINFLAMMATORY_RESPONSE	7.87E-04	2.34E-02	0.48	-0.62	-1.82	38
GOBP_PHOTORECEPTOR_CELL_DIFFERENTIATION	8.10E-04	2.36E-02	0.48	0.54	1.73	59
GOBP_WATER_HOMEOSTASIS	8.35E-04	2.41E-02	0.48	0.54	1.76	61
GOBP_REGULATION_OF_RECEPTOR_SIGNALING_PATHWAY_VIA_STAT	8.68E-04	2.46E-02	0.48	-0.52	-1.77	82

GOBP_NEGATIVE_REGULATION_OF_TUMOR_NECROSIS_FACTOR_SUPERFAMILY_CYTOKINE_PRODUCTION	8.90E-04	2.48E-02	0.48	-0.53	-1.73	60
GOBP_REGULATION_OF_B_CELL_PROLIFERATION	1.02E-03	2.74E-02	0.46	-0.53	-1.74	62
GOBP_REGULATION_OF_MACROPHAGE_ACTIVATION	1.07E-03	2.77E-02	0.46	-0.55	-1.78	57
GOBP_CYTOKINE_PRODUCTION_INVOLVED_IN_INFLAMMATORY_RESPONSE	1.07E-03	2.77E-02	0.46	-0.58	-1.81	49
GOBP_REGULATION_OF_ANTIGEN_PROCESSING_AND_PRESENTATION	1.05E-03	2.77E-02	0.46	-0.78	-1.86	15
GOBP_POSITIVE_REGULATION_OF_ACUTE_INFLAMMATORY_RESPONSE	1.06E-03	2.77E-02	0.46	-0.69	-1.87	25
GOBP_TETRAPYRROLE_BIOSYNTHETIC_PROCESS	1.09E-03	2.78E-02	0.46	0.64	1.80	30
GOBP_NEGATIVE_REGULATION_OF_ADAPTIVE_IMMUNE_RESPONSE	1.23E-03	3.04E-02	0.46	-0.56	-1.81	58
GOBP_SULFATION	1.28E-03	3.07E-02	0.46	0.71	1.86	21
GOBP_NEGATIVE_REGULATION_OF_EXOCYTOSIS	1.28E-03	3.07E-02	0.46	-0.62	-1.78	33
GOBP_REGULATION_OF_NITRIC_OXIDE_METABOLIC_PROCESS	1.35E-03	3.17E-02	0.46	-0.54	-1.73	53
GOBP_DEFENSE_RESPONSE_TO_GRAM_POSITIVE_BACTERIUM	1.42E-03	3.28E-02	0.46	-0.50	-1.68	73
GOBP_DETECTION_OF_EXTERNAL_BIOTIC_STIMULUS	1.43E-03	3.29E-02	0.46	-0.66	-1.80	27
GOBP_LENS_FIBER_CELL_DIFFERENTIATION	1.47E-03	3.35E-02	0.46	0.59	1.72	36
GOBP_REGULATION_OF_MYELOID_LEUKOCYTE_MEDIATED_IMMUNITY	1.66E-03	3.65E-02	0.46	-0.54	-1.75	56
GOBP_PHOTOTRANSDUCTION	1.71E-03	3.70E-02	0.46	0.58	1.75	41
GOBP_REGULATION_OF_WATER_LOSS_VIA_SKIN	1.73E-03	3.71E-02	0.46	0.65	1.80	27
GOBP_GLUTAMATE_RECEPTOR_SIGNALING_PATHWAY	1.83E-03	3.84E-02	0.46	-0.56	-1.77	51
GOBP_NEGATIVE_REGULATION_OF_POSTTRANSCRIPTIONAL_GENE_SILENCING	1.89E-03	3.89E-02	0.46	-0.76	-1.83	15
GOBP_ACTIVATION_OF_JANUS_KINASE_ACTIVITY	1.92E-03	3.94E-02	0.46	-0.76	-1.82	15
GOBP_MODIFIED_AMINO_ACID_TRANSPORT	2.04E-03	4.14E-02	0.43	0.61	1.75	33
GOBP_REGULATION_OF_ACUTE_INFLAMMATORY_RESPONSE	2.05E-03	4.14E-02	0.43	-0.57	-1.77	45
GOBP_PIGMENT_BIOSYNTHETIC_PROCESS	2.21E-03	4.29E-02	0.43	0.50	1.62	62
GOBP_NEGATIVE_REGULATION_OF_PRODUCTION_OF_MOLECULAR_MEDIATOR_OF_IMMUNE_RESPONSE	2.23E-03	4.29E-02	0.43	-0.62	-1.82	37
GOBP_ANTIFUNGAL_INNATE_IMMUNE_RESPONSE	2.28E-03	4.36E-02	0.43	-0.71	-1.78	18
GOBP_MICROGLIAL_CELL_ACTIVATION	2.37E-03	4.47E-02	0.43	-0.59	-1.77	41
GOBP_POSITIVE_REGULATION_OF_NITRIC_OXIDE_METABOLIC_PROCESS	2.65E-03	4.80E-02	0.43	-0.58	-1.72	38
GOBP_PHOTORECEPTOR_CELL_MAINTENANCE	2.71E-03	4.89E-02	0.43	0.58	1.72	40
GOBP_TOLERANCE_INDUCTION	2.76E-03	4.96E-02	0.43	-0.61	-1.71	30

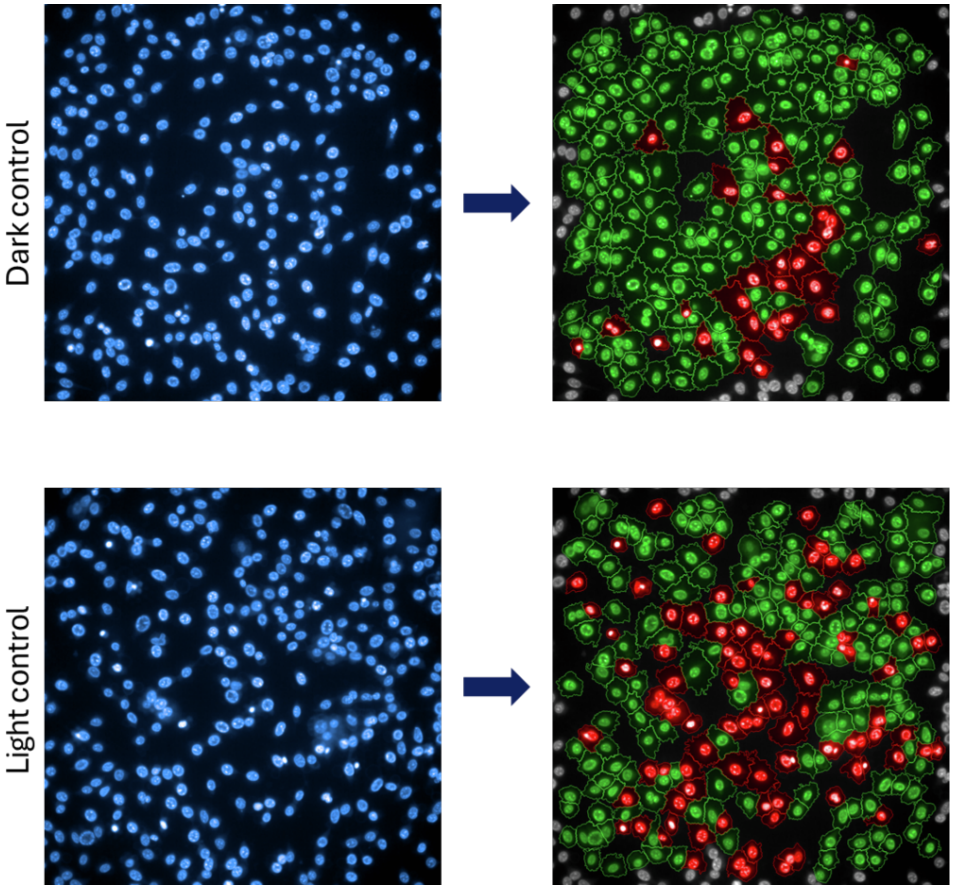

Figure S1: Image automated analysis.

Representative images from Dark and Light controls acquired by the High Content microscope. After Hoechst-dye staining of the nuclei, the automated microscope acquired about 6 pictures per well. The Columbus software detected stained nuclei and characterized each one as ‘live’ (green colour) or ‘dying’ (red colour).

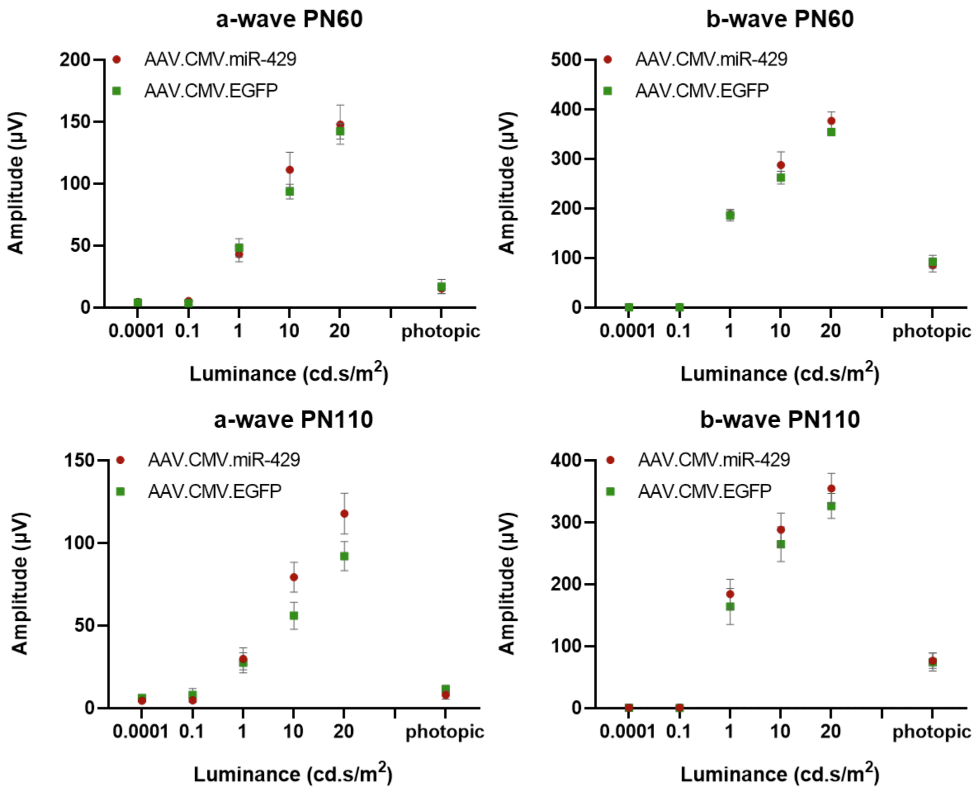

Figure S2: a- and b-wave responses from animals injected at PN30.

ERGs were performed at PN60 (N = 6), and PN110 (N = 7). Data are presented as mean ± SEM.

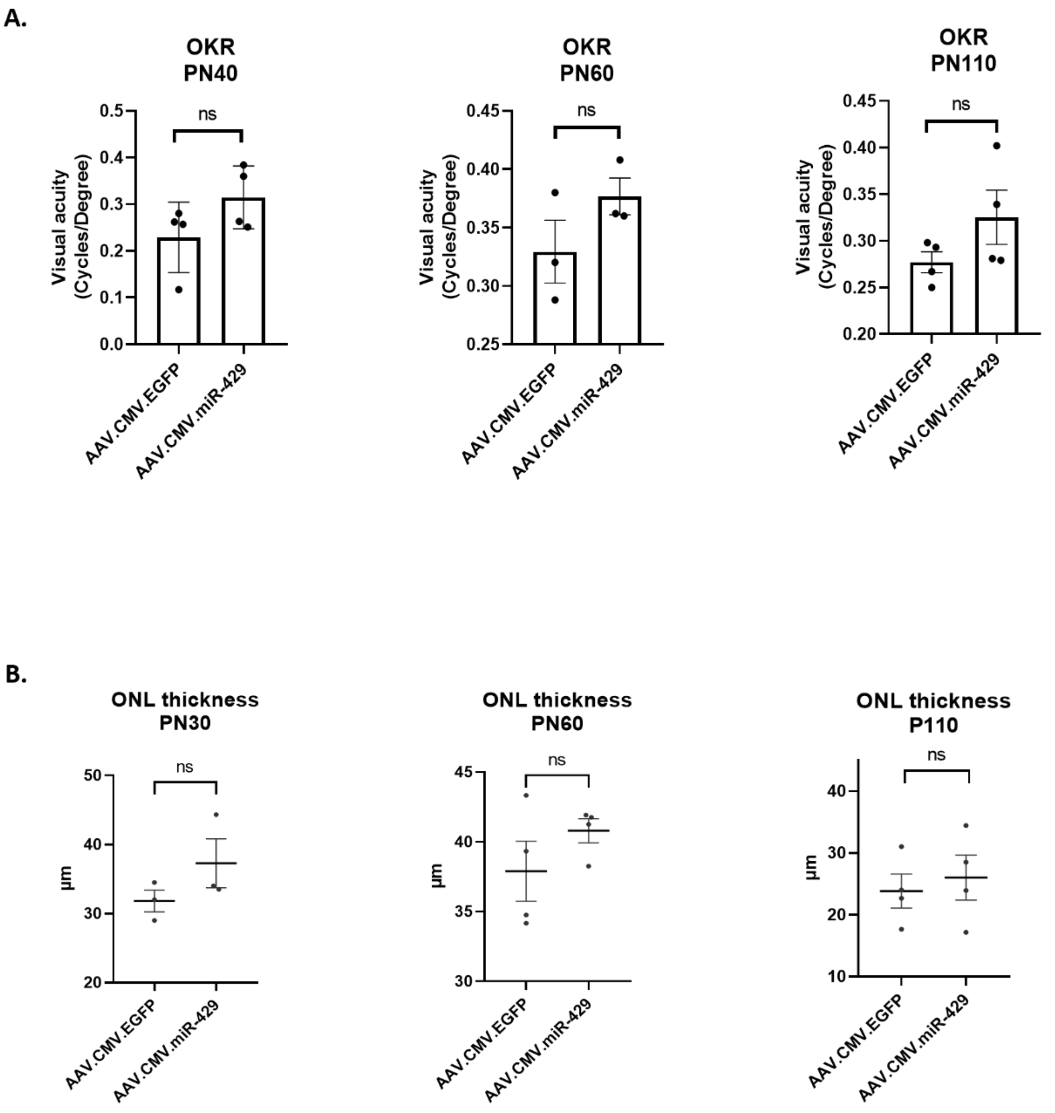

Figure S3: Assessment of visual acuity and retinal morphology in AAV.CMV.miR-429-injected animals.

(A) Optokinetic responses of $Rho^{P23H/+}$ mice injected at PN8 with AAV.CMV.EGFP or AAV.CMV.miR-429 and assayed at PN40 (N = 4), PN60 (N = 3) and PN110 (N = 4). Results are reported as cycles/degree. **(B)** ONL thickness measurements obtained through SD-OCT in $Rho^{P23H/+}$ mice injected at PN8 with AAV.CMV.EGFP or AAV.CMV.miR-429 and assayed at PN30 (N = 3), PN60 (N = 4) and PN110 (N = 4). Data are presented as mean ± SEM. ns: not significant

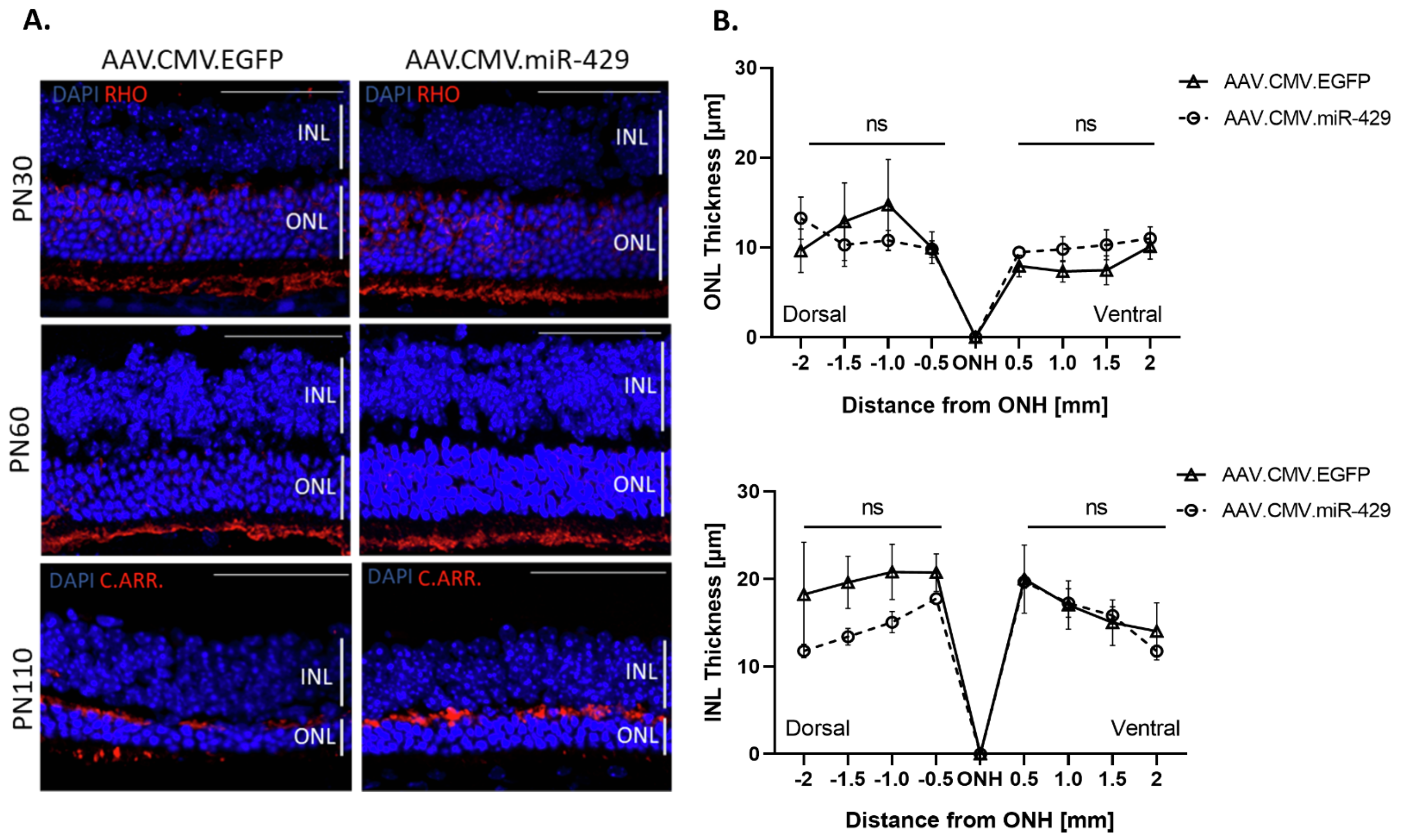

Figure S4: Morphological analysis of AAV.CMV.miR-429-injected eyes.

(A) Immunofluorescence staining for PR markers in Rho$^{P23H/+}$ mice injected at PN8 with AAV.CMV.EGFP or AAV.CMV.miR-429 and assayed at PN30, PN60, and PN110. Rhodopsin (RHO) and Cone Arrestin (C.ARR.) are shown in red. DAPI nuclei counterstaining is shown in blue. INL, inner nuclear layer; ONL, outer nuclear layer. Scale bars: 50 µm. **(B)** Spider graphs showing the ONL and INL thickness of AAV.CMV.miR-429 and AAV.CMV.EGFP-injected retinas at different distances from the optic nerve head (ONH) in the dorsal or ventral retina. AAV.CMV.EGFP. N = 3; AAV.CMV.miR-429. N =4. Data are presented as mean ± SEM. ns: not significant, two-way ANOVA.
