# Supplementary material for: In vitro high-content screening reveals miR-429 as a protective molecule in photoreceptor degeneration
Source: Mol Ther Nucleic Acids. 2024 Dec 22;36(1):102434. doi: 10.1016/j.omtn.2024.102434 (PMC11773019; doi:10.1016/j.omtn.2024.102434)
Supplement: Document S2. Article plus supplemental information [file mmc3.pdf]

Molecular Therapy
Nucleic Acids
Original Article

In vitro high-content screening reveals miR-429 as a protective molecule in photoreceptor degeneration

Georgios Petrogiannakis,[1,2,7] Irene Guadagnino,[1,7] Santiago Negueruela,[1] Martina Di Guida,[1] Elena Marrocco,[1] Mariateresa Pizzo,[1] Annalaura Torella,[1,3] Mariateresa Zanobio,[3] Marianthi Karali,[3,4] Diego Luis Medina,[1,5] Sabrina Carrella,[6] and Sandro Banfi[1,3]

[1]Telethon Institute of Genetics and Medicine (TIGEM), Via Campi Flegrei 34, 80078 Pozzuoli, Italy; [2]Department of Environmental, Biological and Pharmaceutical Science and Technology, University of Campania “Luigi Vanvitelli”, Via Vivaldi 43, 81100 Caserta, Italy; [3]Department of Precision Medicine, University of Campania “Luigi Vanvitelli”, Via Luigi De Crecchio 7, 80138 Naples, Italy; [4]Multidisciplinary Department of Medical, Surgical and Dental Sciences, Eye Clinic, University of Campania “Luigi Vanvitelli”, Via Pansini 5, 80131 Naples, Italy; [5]Department of Medical and Translational Science, Federico II University, 80131 Naples, Italy; [6]Biology and Evolution of Marine Organisms Department, Stazione Zoologica Anton Dohrn, Villa Comunale, 80133 Naples, Italy

Inherited retinal diseases (IRDs) are clinically and genetically heterogeneous disorders characterized by progressive photoreceptor degeneration and irreversible vision loss. MicroRNAs (miRNAs), a class of endogenous non-coding RNAs with post-transcriptional regulatory properties, are known to play a major role in retinal function, both in physiological and pathological conditions. Given their ability to simultaneously modulate multiple molecular pathways, miRNAs represent promising therapeutic tools for disorders with high genetic heterogeneity, such as IRDs. In the present study, we performed high-content imaging (HCI) screening to assess the impact of miRNA overexpression on a photoreceptor cell line undergoing light-induced degeneration. More than 1,200 miRNAs were assayed for putative protective effects in light-stressed 661W photoreceptor-like cells, and the top-performing miRNAs were further validated in independent *in vitro* assays. miR-429 showed the strongest cell-protective effect *in vitro*. Adeno-associated viral vector-mediated subretinal delivery of miR-429 in the *Rho*$^{P23H/+}$ IRD mouse model preserved electrophysiological responses and was associated with reduced inflammatory processes in the retina. We demonstrate that the HCI *in vitro* assay we devised is a reliable screening method to select candidate molecules for mutation-independent therapeutic approaches for retinal disorders. Moreover, our data indicate that miR-429 represents a potential therapeutic target against photoreceptor degeneration.

INTRODUCTION

Inherited retinal diseases (IRDs) are a heterogeneous group of rare monogenic diseases caused by mutations in over 280 genes (http://sph.uth.edu/RETNET/, July 2024). These disorders are characterized by progressive degeneration of photoreceptor cells (PRs). PRs are the sensory neuron cells localized in the outer retina responsible for the conversion of light energy into membrane potential changes in the phototransduction cascade.[1] Their dysfunction and/or death causes irreversible vision loss and represents the main cause of childhood and adult blindness in industrialized countries.[2] The pattern of PR degeneration varies across different clinical subtypes of IRDs. It can either start from rods and extend to cones (e.g., in the case of retinitis pigmentosa [RP]), or vice versa (e.g., in cone-rod dystrophies), or it may exclusively affect cones (e.g., in cone dystrophies).[3,4] The exact mechanisms underlying PR dysfunction and degeneration are poorly understood. Nevertheless, some of the molecular pathways involved in PR death have been identified thanks to studies on animal models of IRDs. These pathways include oxidative stress, inflammation, Ca^{2+} imbalance, endoplasmic reticulum (ER) stress responses, and high levels of cyclic guanosine monophosphate (cGMP), among others.[5]

Considering the high prevalence of IRDs in the population (collectively affecting about 1 in 2,000 individuals)[6,7] and the importance of protecting PRs from death to counteract the associated vision loss, there is a strong interest in developing effective treatments that can delay or ideally prevent PR degeneration. Gene therapy approaches are considered powerful tools to tackle these conditions. However, despite tremendous effort over the past 25 years, only one such approach, based on a gene-replacement strategy, has been approved for clinical use.[8] The high genetic heterogeneity of IRDs is a further obstacle to developing gene-specific treatments for each clinical form within a reasonable time frame. As many IRDs share common pathogenic events downstream of the primary genetic defect, such as mitochondrial dysfunction, glycolytic deregulation,

Received 23 July 2024; accepted 18 December 2024;
https://doi.org/10.1016/j.omtn.2024.102434.

[7]These authors contributed equally

Correspondence: Sandro Banfi, Telethon Institute of Genetics and Medicine (TIGEM), Via Campi Flegrei 34, 80078 Pozzuoli, Italy.
E-mail: banfi@tigem.it

Published by Elsevier Inc. on behalf of The American Society of Gene and Cell Therapy.
This is an open access article under the CC BY license (http://creativecommons.org/licenses/by/4.0/).

and microglia activation,[9,10] alternative therapeutic strategies are being investigated to tackle PR degeneration processes in a mutation-independent manner.[11]

In the past few years, microRNAs (miRNAs) have emerged as important regulatory elements in retinal pathophysiology.[12–15] These small, non-coding RNA molecules act as post-transcriptional gene expression regulators in a sequence-specific manner and simultaneously modulate several molecular pathways, rendering them promising therapeutic targets in genetically heterogeneous diseases, such as IRDs. In fact, the protective effect of the modulation of some miRNAs in PR degeneration progression has already been reported for miR-204, miR-181a/b, and miR-6937.[16–18] An unbiased, systematic evaluation of the modulation of all known miRNAs in PR degeneration could yield additional candidates. However, the paucity of reliable *in vitro* models, which would be highly practical for a primary screen of the entire catalog of miRNAs in a systematic and efficient manner, is an important limitation.

The 661W cell line, a transformed murine PR line derived from a retinal tumor that expresses several cone markers, was previously proposed as a useful PR-like system.[19,20] Interestingly, 661W cells are susceptible to photo-oxidative damage,[21] which is one of the major causes of PR degeneration in IRDs.[22,23] This system has already been used as a model for studying retinal diseases, also through high-throughput approaches.[24–26] Hence, we decided to exploit this model in a cell-based high-content imaging (HCI) setting to systematically screen a human miRNA library and identify miRNAs that impact PR degeneration. We found that transfection of a miR-429 mimic exerted significant cell protection, and therefore, we studied its effects *in vivo*. Overexpression of miR-429 in the retina of an RP mouse model using adeno-associated viral (AAV) vector delivery preserved retinal functionality. Transcriptomic and immunohistological analyses indicated that miR-429 overexpression is associated with the attenuation of inflammation-related biological processes in the retina, suggesting it can delay PR dysfunction and/or degeneration.

RESULTS

Impact of miRNAs on light-stressed PRs: A cell-based HCI screening assay

To systematically test the impact of miRNAs on PR death, we carried out an HCI analysis of 661W cells exposed to light damage.[21] A schematic representation of the experimental setup is shown in Figure 1A. Following light exposure (see the materials and methods for details), nuclei were stained with Hoechst 33342 and visualized under a high-content microscope to calculate the percentage of dying cells in each well.[27–29] Although Hoechst 33342 stains all cells, it is possible to discriminate between healthy and dying ones based on differences in fluorescence intensity and nuclear morphology (Figure S1). Each 384-well plate contained two types of controls, namely "dark" controls (i.e., wells covered with non-transparent tape to prevent light exposure) and "light" controls (i.e., wells exposed to light) (Figure 1B). Cells in control wells were not transfected with any miRNAs but were used to assess whether the light stress applied to each plate was sufficient for effective screening. In other words, detecting increased cell death in "light" controls compared to the "dark" ones served as an indication that the results of each screening experiment could be considered reliable. In addition, some of the control wells in each plate contained cells transfected with a red fluorescent dye (Dy547)-labeled mimic to confirm efficient transfection.

Using the above-described procedure, 1,268 miRNAs (listed in Table S1) were screened for their impact on the viability of 661W cells after light exposure (Figure 1C). To assess the effect of each miRNA on cell viability, the percentage of dying cells in each well was normalized with respect to the light controls in the same plate, and Z scores were calculated. We then determined the average Z score of three biological replicates for each miRNA (Table S1) and ranked them from lowest to highest (Figure 1C). miRNAs with the lowest Z score values were those conferring the strongest cell protection, and vice versa. We decided to use as cutoff Z score values < -2.1. To ensure the reliability of our further analyses, we chose to proceed only with the miRNAs present in MirGeneDB (Table S1), a database containing miRNAs that are more likely to represent *bona fide* entities based on their genomic features.[30,31] Finally, we ranked the remaining miRNAs based on their coefficient of variation (CV) values to exclude from further validation those displaying excessive variability across the three replicates analyzed. As a result, the top six miRNAs identified as being protective were selected for further investigation (Figure 1D).

Secondary *in vitro* evaluation of the cell-protective miRNAs

After the primary HCI screen, the protective effect of the six identified miRNAs was tested with a secondary *in vitro* assay (Figure 2A). For this assay, cells were cultured and transfected with miRNA mimics in 12-well plates. Due to the different plate format, we detected a notable decrease in cell viability already after 3 h of light-emitting diode (LED) light stress and adjusted the duration of light exposure accordingly. Unlike the primary screen, cell viability was determined by staining with trypan blue dye, and the percentage of cell death was calculated manually. This low-throughput configuration offered the possibility to test the impact of each transfected miRNA in both light and dark conditions simultaneously, allowing us to calculate cell viability in either setting.

When we compared miRNA-transfected cells with control cells (transfected with the negative mimic) after light exposure, we found that five out of the six selected miRNAs led to reduced cell death levels. Specifically, transfection of miR-150-3p, miR-371a-5p, miR-429, miR-514b-5p, and miR-5010-5p correlated with fewer dying cells, while in the case of miR-346, the extent of cell death was similar to its control. Out of the five miRNAs that showed protection, only miR-429 increased cell viability in a statistically significant manner (Figure 2B). This finding was consistent with the results of the primary screen where miR-429 had the lowest Z score value, indicative of the strongest protective effect. Taken together, the results of the secondary assay corroborate the robustness and validity of the primary high-throughput screen by confirming the protective action of the short-listed miRNAs on 661W cells undergoing light-induced damage.

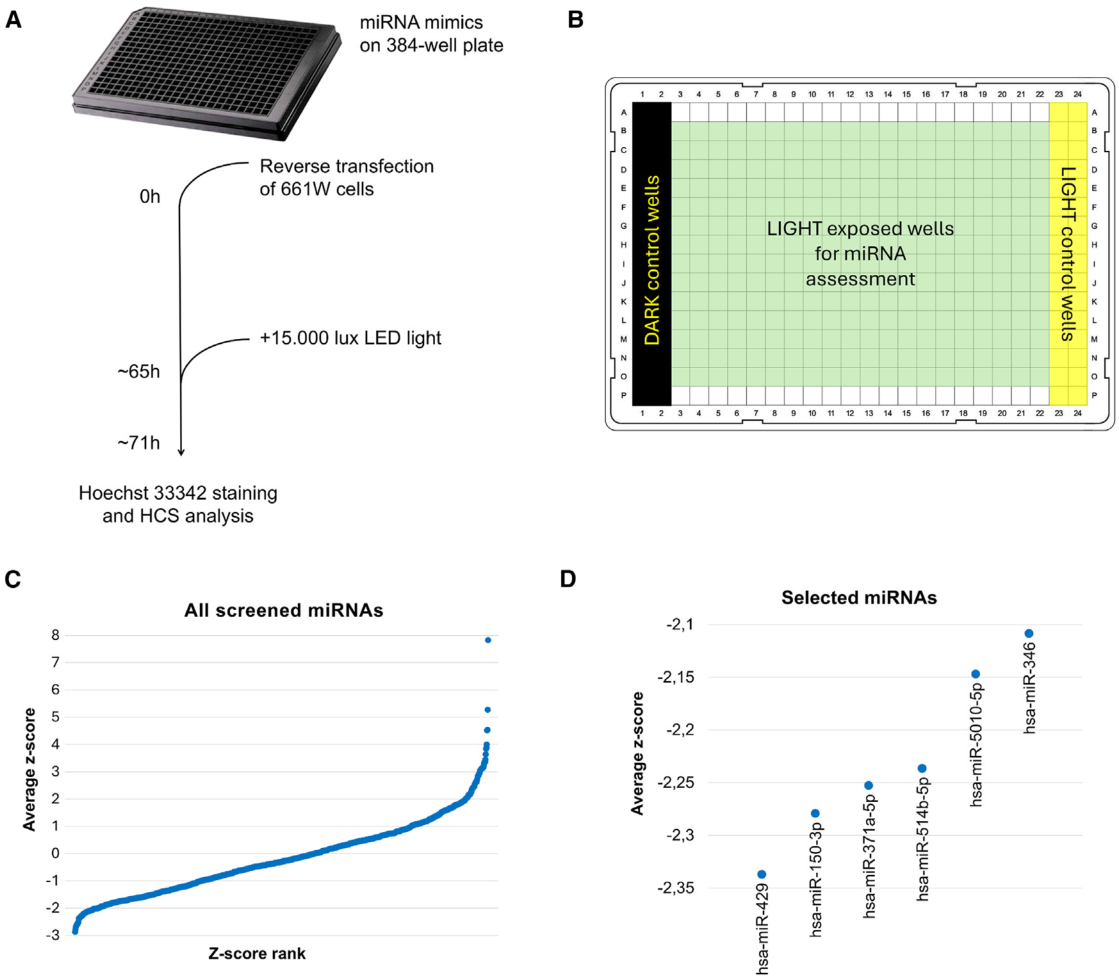

Figure 1. miRNA-based HCI screening in light-stressed 661W cells

(A) Schematic representation of the experimental workflow. (B) Scheme of sample distribution in each 384-well plate. Dark and light control cells were plated in the first two and last two columns, respectively (black and yellow colored areas). (C) Average *Z* score derived from each of the 1,268 screened miRNAs (n = 3 biological replicates). (D) Cell-protective miRNAs that fulfilled the filtering criteria and were selected for further investigation.

AAV-mediated subretinal delivery of miR-429 to the retina is effective and does not induce functional alterations

Based on the *in vitro* evidence that suggested a protective role of miR-429 overexpression against light-induced 661W degeneration, we tested its effect *in vivo* by overexpressing the miR-429 precursor sequence (pre-miR-429) in the retina of a mouse IRD model. To this end, we opted for an AAV vector-mediated delivery using the AAV8 serotype, which is reported to efficiently transduce the retinal pigment epithelium (RPE) and PRs upon subretinal administration in several species, including mice.[32] The human pre-miR-429 gene was cloned initially in an AAV plasmid vector downstream of the constitutive cytomegalovirus (CMV) promoter (pAAV.CMV.pre-miR-429) (Figure 3A). This plasmid was then used to generate recombinant AAV8 vectors expressing pre-miR-429 (AAV.CMV.miR-429). To confirm effective overexpression and processing of miR-429 in the mouse retina, particularly in PRs, C57BL/6J wild-type (WT) mice (n = 4) were subretinally injected with the AAV.CMV.miR-429 vector at postnatal day (P)8. As controls, the contralateral eyes were injected with the AAV.CMV.EGFP, a vector expressing the enhanced green fluorescent protein (EGFP) reporter. Please note that the injection of AAV.CMV.miR-429 contained also 1/10 of AAV.CMV.EGFP virus. The latter co-injection was carried out to indirectly monitor AAV.CMV.miR-429 distribution in injected retinas. At P30, we quantified the expression levels of the mature form of miR-429 in total RNA from whole eyes and observed the proper processing of the exogenously provided construct in AAV.CMV.miR-429-injected eyes (Figure 3B).

We then assessed whether miR-429 delivery induces deleterious effects on healthy retinas. WT mice at P8 were subretinally injected with the AAV.CMV.miR-429 vector in one eye and the AAV.CMV.EGFP control vector in the contralateral eye. Retinal function was then tested by

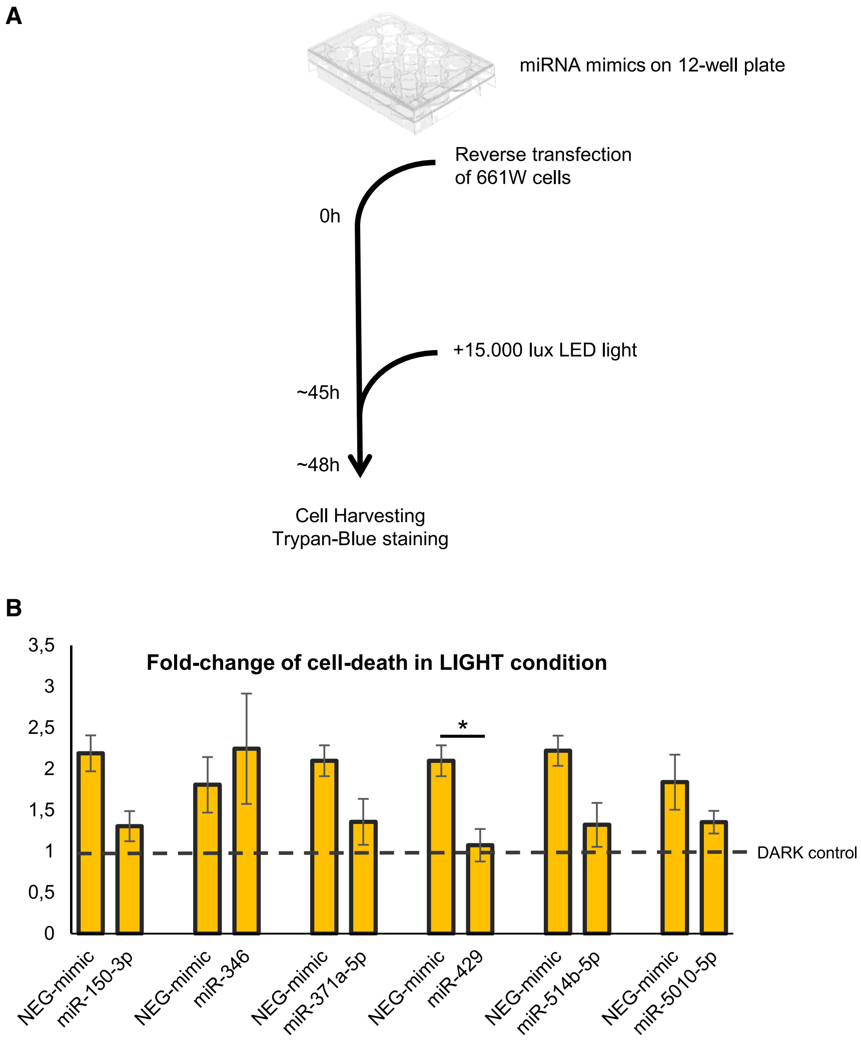

Figure 2. Secondary cell viability *in vitro* assay for validation of the selected cell-protective miRNAs

(A) Schematic representation of the experimental workflow. (B) Cell viability assay after 3 h of light exposure. The extent of cell death, calculated based on trypan blue staining, is shown as a fold change in light conditions after the normalization of each sample against its corresponding dark condition ($n = 3$). The fold change of each miRNA is plotted next to its own control transfected with the negative mimic (NEG mimic) and similarly normalized. Data are presented as mean ± SEM. $^{*}p = 0.024$, two-way ANOVA.

full-field electroretinogram (ERG) at P30, P60, and P110. We did not detect any significant differences between miR-429- and contralateral control-injected eyes at any of the three stages (Figure 3C), suggesting that the delivery of miR-429 to healthy retinas does not cause evident signs of retinal dysfunction.

Subretinal injection of pre-miR-429 preserves visual function in an IRD mouse model

To test if miR-429 could confer protection against PR degeneration *in vivo*, we applied the same strategy in a mouse model of IRD. Specifically, we used the *Rho*$^{P23H/+}$ knockin mouse, a well-characterized model that carries the most common point mutation in the rhodopsin gene responsible for autosomal dominant RP.[33,34] The *Rho*$^{P23H/+}$ knockin mouse develops retinal degeneration that mirrors the RP phenotype of patients carrying the orthologous variant.[33,35] To explore whether miR-429 overexpression could impact IRD progression, we injected AAV.CMV.miR-429 at three postnatal time points: (1) at P8, when the mouse retina is not completely differentiated and its degeneration has not started yet, (2) at P20, which corresponds to the peak of the degenerative process,[36] and (3) at P30, a more advanced stage when 60%–70% of PRs are still preserved in this animal model.[36] At all time points, the contralateral (control) eye was subretinally injected with AAV.CMV.EGFP (see also the materials and methods). Retinal function was then recorded by ERG at P30, P60, and P110 for mice injected at P8 and at P60 and P110 when injections were performed at P20 or P30.

We observed a statistically significant improvement of the scotopic a- and b-wave amplitudes at all three stages of analysis (i.e., P30, P60, and P110) in eyes injected with the AAV.CMV.miR-429 vector at P8 compared to the contralateral controls (Figure 4A). In mice injected at P20, miR-429-treated eyes showed significantly higher scotopic b-wave ERG responses at P60 and a significant amelioration in both a- and b-waves at P110 when compared to controls (Figure 4B). When the injection was performed at P30, we did not detect any statistically significant improvement in ERG responses except for a tendency toward protection in a- and b-waves at P110 (Figure S2). We then decided to assess visual acuity in miR-429-injected eyes. To achieve this goal, we carried out an optokinetic response (OKR) in mice injected at P8, i.e., the injection time point showing the highest protective effect. This analysis revealed that miR-429-treated eyes displayed no statistically significant difference in OKRs vs. control-treated eyes (Figure S3A). By immunofluorescence analysis, we performed staining with PR markers as well as quantification of outer nuclear layer (ONL) and inner nuclear layer (INL) thicknesses, but we did not observe any major morphological differences between miR-429- and GFP-injected retinas (Figure S4). Finally, analysis of the ONL with spectral domain-optical coherence tomography (SD-OCT) showed no statistically significant increases of ONL thickness in miR-429-injected eyes (Figure S3B).

Overall, these data indicate that the overexpression of miR-429 at the early stages of degeneration can preserve retinal function in the *Rho*$^{P23H/+}$ knockin mouse model.

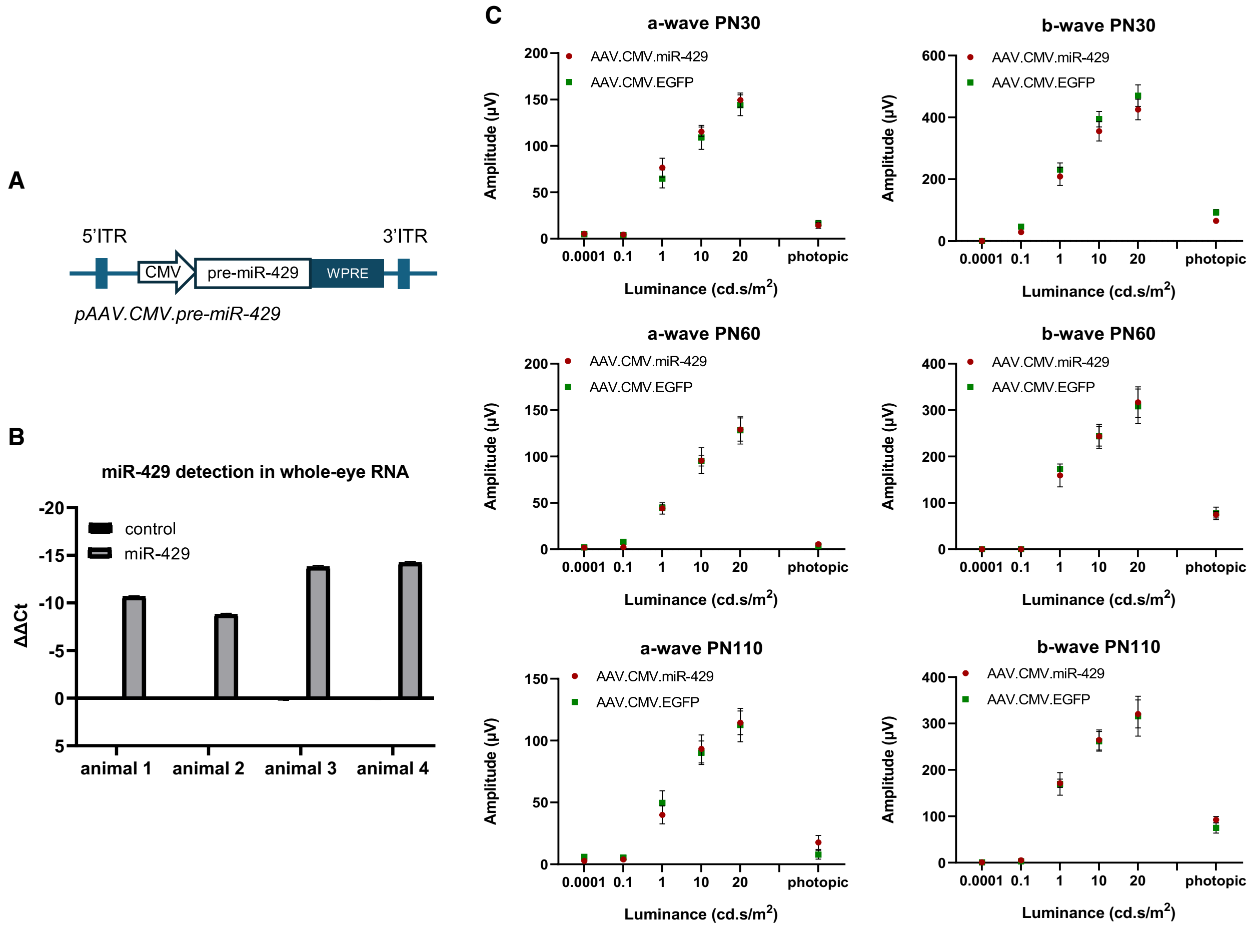

Figure 3. Design of the AAV vector and the assessment of the processing and effect on retinal function of miR-429 overexpression in WT mouse retina

(A) Schematic representation of the expression cassette (pAAV.CMV.pre-miR-429) used for AAV.CMV.miR-429 vector production. (B) Detection of miR-429 in whole-eye RNA. The results are shown as ΔΔCt values. miR-429 was overexpressed in the eyes of all four animals injected with the AAV.CMV.miR-429 vector. Contralateral control eyes were injected with AAV.CMV.EGFP (control). (C) a- and b-wave ERG analysis for the effect of miR-429 overexpression in WT mice. WT eyes injected at P8 with the AAV.CMV.miR-429 and the control vector (AAV.CMV.EGFP) did not show significant differences in retinal function at any of three time points of analysis (P30: AAV.CMV.miR-429 $n = 9$, AAV.CMV.EGFP $n = 8$; P60 and P110: AAV.CMV.miR-429 $n = 4$, AAV.CMV.EGFP $n = 3$). Data are presented as mean ± SEM.

Transcriptomic analysis reveals biological processes modulated by miR-429 delivery in the retina

To gain insight into the molecular mechanisms that are involved in the protective effects of AAV.CMV.miR-429 administration to the retina, we carried out a comparative transcriptomic analysis by RNA-seq. *Rho*$^{P23H/+}$ mice ($n = 7$) were injected at P8 using the above-mentioned scheme, i.e., one eye with AAV.CMV.miR-429 and the contralateral eye with AAV.CMV.EGFP as a control. Total RNA was isolated from optic cups at P30, a stage at which the degeneration in this model is ongoing, with 60%–70% of PRs still viable.[36]

Bioinformatics analysis did not reveal differentially expressed genes of interest according to the established threshold (data not shown). However, miRNAs can modulate biological processes by acting on multiple transcripts in a subtle but impactful manner.[37] To identify such mild regulatory effects, we performed a gene set enrichment analysis (GSEA). Two main categories were identified among the enriched biological processes in response to miR-429 overexpression (Figure 5A; Table S2). First, processes related to visual functions, such as PR maintenance and light stimulus detection, were positively enriched. Secondly, we obtained many terms with negative enrichment associated with immune and inflammatory responses. An unrestrained inflammatory response has often been associated with PR loss in multiple IRD models,[38] including models of the P23H rhodopsin mutation.[39,40]

Given the enrichment for innate immunity and inflammation-related terms among the downregulated biological processes, we investigated whether miR-429 overexpression modulates microglia activation in the retina. We assessed microglia activation in *Rho*$^{P23H/+}$ mice at

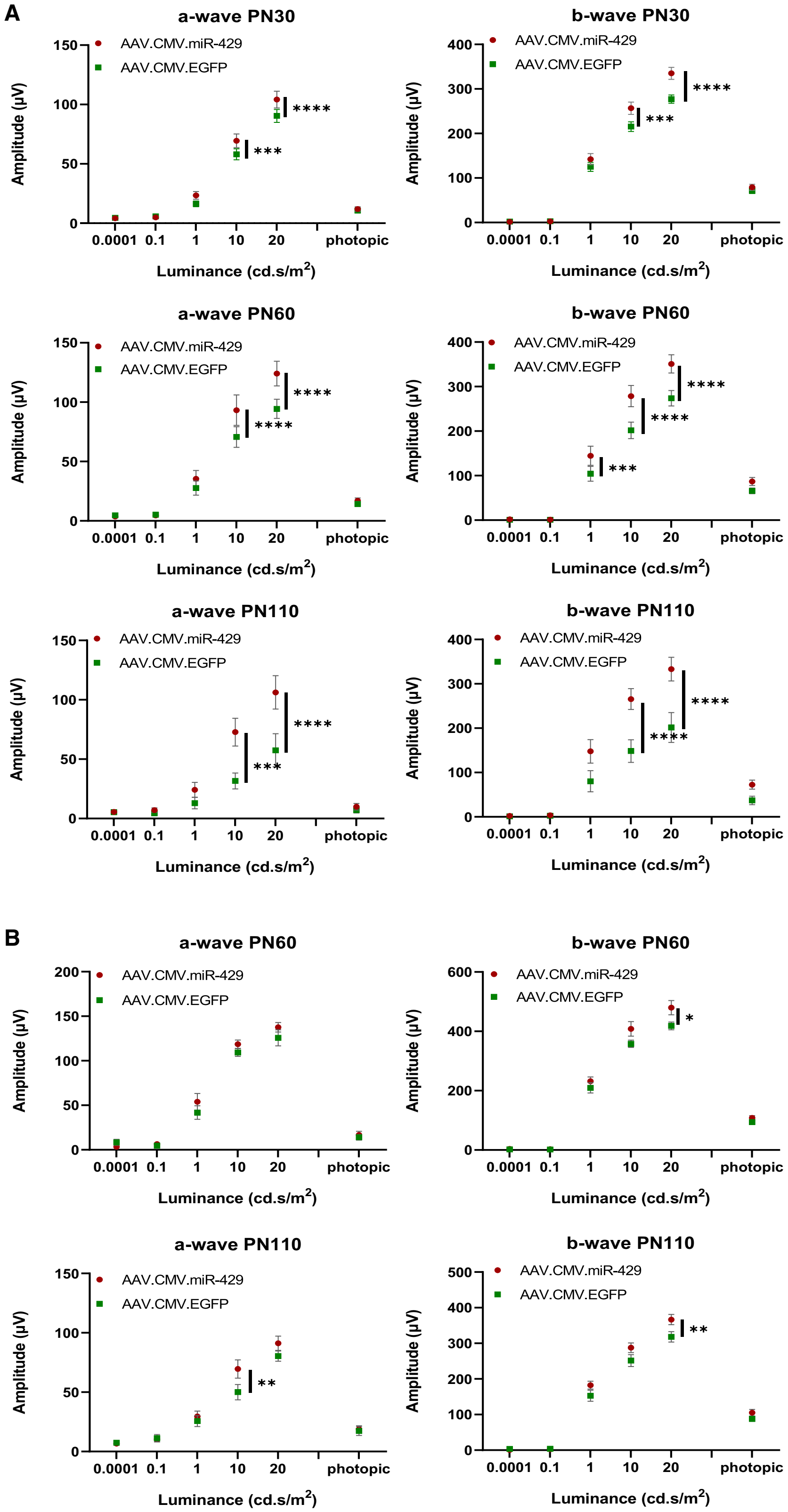

Figure 4. AAV-mediated delivery of miR-429 improves ERG responses in *Rho*$^{P23H/+}$ mice

(A) a- and b-wave responses from animals injected at P8. ERGs were performed at P30 (n = 29), P60 (n = 22), and P110 (n = 12). (B) a- and b-wave responses from animals injected at P20. ERGs were performed at P60 (n = 12) and P110 (n = 10). Data are presented as mean ± SEM. $^*p < 0.05$, $^{**}p < 0.01$, $^{***}p < 0.001$, and $^{****}p < 0.0001$, two-way ANOVA/mixed-effects analysis.

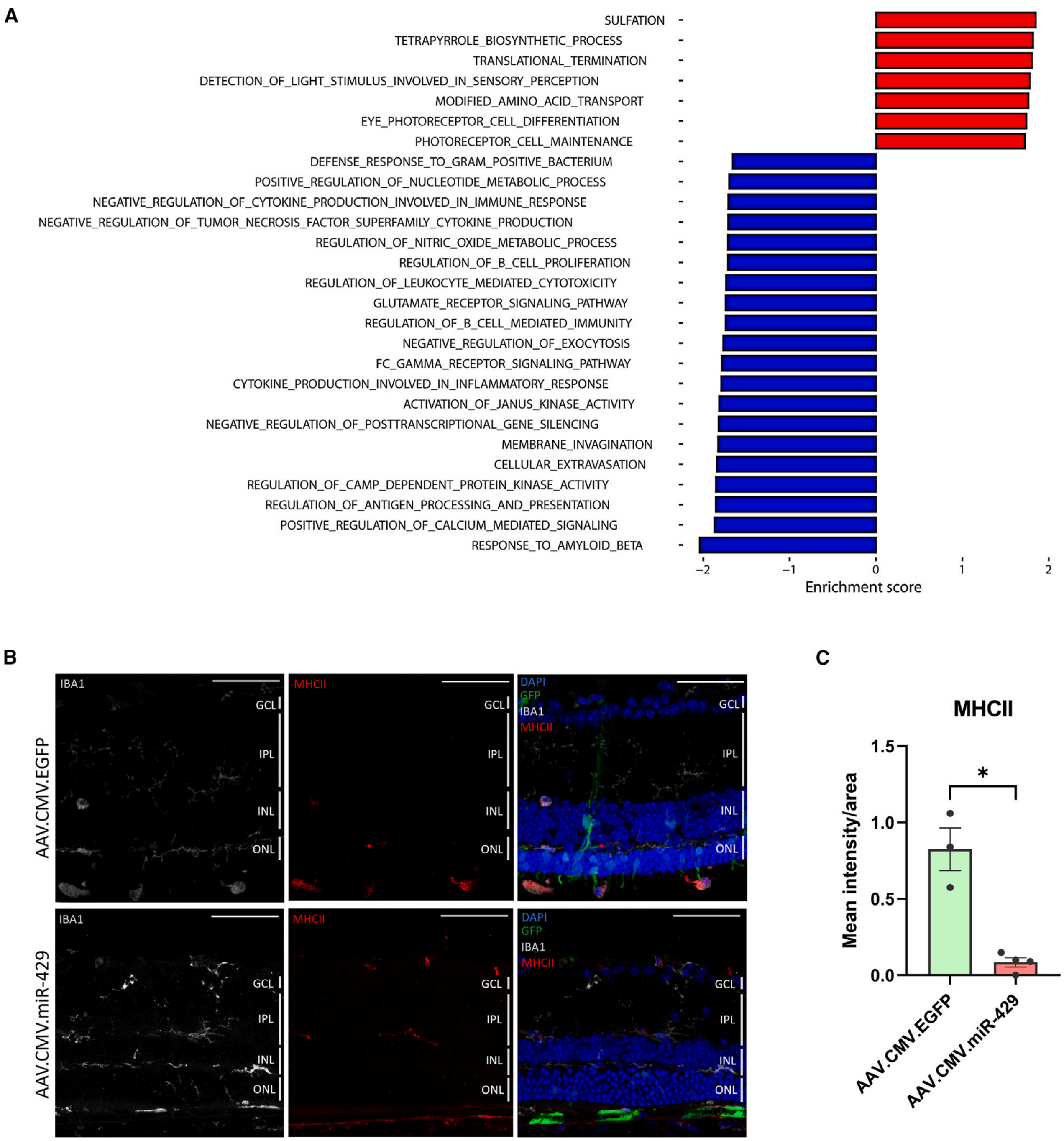

Figure 5. AAV-mediated delivery of miR-429 in *Rho*$^{P23H/+}$ mice is associated with attenuation of microglia activation

(A) GO enrichment analysis derived RNA-seq data from optic cups. All biological processes shown in the graph were significantly altered by AAV.CMV.miR-429 injections compared to the GFP-injected controls. Red bars, upregulated terms; blue bars, downregulated terms. (B) Representative immunofluorescence images of P30 retinal sections from *Rho*$^{P23H/+}$ mice injected with AAV.CMV.miR-429 or AAV.CMV.EGFP at P8 stained for the microglia marker Iba1 (gray) and MHC class II (red) (n = 4). DAPI nuclei counterstaining is shown in blue. GCL, ganglion cell layer; INL, inner nuclear layer; IPL, inner plexiform layer; ONL, outer nuclear layer. Scale bars: 50 μm. (C) Fluorescence quantification of MHC class II staining. Data are represented as mean intensity/area. AAV.CMV.EGFP n = 3; AAV.CMV.miR-429 n = 4. *p < 0.05, Welch's t test.

the same time point used for the transcriptomic studies (i.e., in eyes injected at P8 and enucleated at P30) by double immunostaining using antibodies against ionized calcium-binding adaptor molecule 1 (IBA1) and major histocompatibility complex (MHC) class II (Figures 5B and 5C). Iba1 is a marker of both quiescent and activated microglia, while MHC class II expression is associated with microglial cell activation. Indeed, in miR-429-injected retinas, fewer microglial cells transformed to an ameboid morphology in the subretinal area, and there were more resting microglia displaying the typical steady-state ramified form. Furthermore, we detected decreased immunostaining for MHC class II, a marker of activated (phagocytic) microglia (Figures 5B and 5C). Taken together, these data indicate that miR-429 delivery to the retina of *Rho*$^{P23H/+}$ mice is associated with suppression of microglia activation and inflammation, possibly contributing to the amelioration of PR function.

DISCUSSION

Approximately, one-fifth of all known miRNAs are expressed in the retina,[41] and a limited subset of them have been shown to be essential for retinal development and function.[42,43] It is therefore not surprising that the dysregulation of miRNAs is linked to various retinal degenerative diseases and developmental genetic disorders.[44] Considering also the protective effect of miRNA modulation in *in vivo* models of retinal disease,[16,17] we performed a high-throughput *in vitro* screen to assess the impact of miRNAs on PR degeneration. To achieve this systematic approach, we set up a cell-based HCI protocol suitable for effective screening of miRNA libraries in 661W cells. Light exposure on 661W cells represents a widely used system to study PR degeneration because the pathways activated in this system, such as oxidative, mitochondrial, and ER stress, are known to play a pathogenic role in degenerative diseases affecting the human retina.[45,46] This study represents the first high-throughput scale screening using this light-induced stress system for miRNA characterization. We identified six miRNAs that, when transfected in 661W cells, conferred protection from light-induced damage. The reliability of the HCI approach was corroborated through a secondary *in vitro* assessment of the identified cell-protective miRNAs and by the outcome of the *in vivo* analysis performed in the *Rho*$^{P23H/+}$ mouse model.

miR-429 was the top-scoring positive hit from the primary screening and was clearly validated in the secondary *in vitro* test. This miRNA belongs to the miR-200 family,[47] which is highly conserved during deuterostome evolution and present across all vertebrate classes.[48] The family consists of a single ortholog in the fruit fly (miR-8) and five members in vertebrates, including miR-429.[49] The miR-8/miR-200 family has been reported to have a role in the regulation of neurogenesis and gliogenesis in the developing central nervous system of invertebrates and vertebrates.[49] It has also been described as having a role in different forms of cancer.[50] To date, however, there is limited information about a role for miR-429 in retinal function. Small RNA-seq analysis of healthy human retinas showed that miR-429 is expressed at low levels.[41] Moreover, recently published data indicated that miR-429 negatively regulates the heparanase-VEGF pathway and might play an important role in the progression of hypoxia-induced retinal neovascularization.[51]

Besides its protective effect on PRs *in vitro*, miR-429 overexpression preserved retinal function in an *in vivo* model of autosomal dominant RP, as assessed by AAV-mediated delivery to the retina of the *Rho*$^{P23H/+}$ knockin mouse. Importantly, we observed improved ERG responses in retinas injected not only at P8 but also at P20, a time point when the degenerative process has reached its peak.[36,52] This finding, along with the initial safety data, is important in terms of the translational value and clinical applicability of the approach because it shows that miR-429 administration is effective also at time points and disease stages that are clinically relevant. However, this slight functional preservation after disease onset may be relevant, as it might support any clinical potential of our approach.

miR-429 administration at P8 was accompanied by the best results in terms of ERG response, as opposed to later injection time points (P20 and P30) (Figures 4 and S2). The reduction in the protective effect at later injection time points can be explained by both more advanced PR damage and the latency in the expression of the AAV.CMV.miR-429 vector. It has been reported that the first signs of PR loss in the *Rho*$^{P23H/+}$ mouse model are already evident at P15.[53] From that time point onward, the degeneration progresses quickly, reaching a peak during the third week of age, and by P30, about 60%–70% of the total number of PRs remain.[36,52] As a consequence, subretinal injections during this phase of degeneration, as well as the time frame needed for efficient AAV-mediated transgene expression,[54,55] results in reduced improvement of visual function, as more PRs are irreversibly damaged or lost by then. Finally, it is known that miRNAs can exert their action in a non-cell-autonomous manner via their secretion in extracellular vesicles (EVs).[56,57] Our preliminary data indicate that indeed, following AAV-mediated subretinal delivery, miR-429 is present in retinal EVs (data not shown). Therefore, it is tempting to speculate that the best protective effects observed after P8 injection may also be explained by a more efficient inclusion in EVs. Obviously, further experiments are necessary to gain further insight into the time frame of miR-429 protection.

It is interesting to underline that through an *in vitro* screening based on light-induced damage, we were able to identify a molecule that exerts a protective role also in an IRD model due to a mutation that causes rhodopsin protein misfolding.[34] These results further support the validity of the 661W *in vitro* assay as a surrogate model of PR degeneration with a Mendelian cause. Indeed, among the pathways that play a role in the pathogenesis of IRDs, photo-oxidative damage is among the most relevant ones.[21,23]

Our *in vivo* findings support other studies reporting preserved retinal function after miRNA modulation in retinas of IRD models.[16–18] For instance, we and others showed that the downregulation of miR-181a/b in the retina is associated with improved retinal morphology and visual function in different murine IRD models with either dominant or recessive inheritance.[16,58] Moreover, we demonstrated that

the AAV-mediated administration of miR-204 in the retina of two IRD models (*RHO*-P347S and *Aipl1*$^{-/-}$) slows down PR degeneration.[17] This evidence thus highlights the potential of miRNAs as therapeutic tools for mutation/gene-independent treatment of IRDs and possibly other genetically heterogeneous diseases. Nevertheless, to demonstrate the clinical value of the miR-429 delivery, it will be necessary to extend the study to additional IRD models, consolidate its safety profile in WT animals, and dissect the molecular mechanisms underlying the observed protective effect.

Our transcriptomic data revealed a downregulation of terms related to inflammation and innate immune response in miR-429-injected eyes (Figure 5A), suggesting that at least part of the protective action of miR-429 overexpression is exerted by dampening microglial activation in the retina, as indicated also by immunofluorescence assays (Figure 5B). The dissection of the molecular mechanisms through which miR-429 overexpression modulates, either in a direct or in an indirect manner, microglia activation remains to be elucidated. Nevertheless, it is well established that prolonged or excessive microglial activation induces inflammatory responses that exacerbate retinal degeneration due to persistent proinflammatory signals, causing the phagocytosis of even non-apoptotic PRs.[10] Indeed, understanding the relationship between microglia activation and neurodegenerative disease progression is currently a topic of strong interest due to its potential therapeutic applications.[59] It is difficult to treat or cure inherited retinal diseases, especially when PRs are affected and irreversibly lost. Currently, there is a strong need for reliable, yet amenable, models that recapitulate PR degeneration to allow fast and efficient testing of new neuroprotective treatments. In that respect, *in vitro* models of PR degeneration are particularly promising. Here, we devised a high-throughput method that was successful in identifying miRNAs that counteract PR death upon light-induced damage. *In vivo* validation experiments, such as those performed for miR-429, should now be applied to the other shortlisted miRNAs of the primary screen. For the purpose of this study, we focused on cell-protective miRNAs, but the primary screen also revealed miRNAs that, upon overexpression, exacerbate PR death (see miRNAs on the right side of the plot in Figure 1C, with Z scores > 2.1). Future studies should be designed to downregulate these miRNAs *in vivo* and evaluate their capacity to slow down degeneration. In conclusion, this study confirms the effectiveness of the primary *in vitro* HCI screening method to gain insights into the undoubtedly key role of miRNAs in PR degeneration or survival. We expect that the described approach will facilitate large-scale screenings for the development of novel mutation/gene-independent therapeutic strategies to counteract PR degeneration.

MATERIALS AND METHODS

HCI screening assay

HCI screening was employed to identify miRNAs that can protect 661W cells from light-induced degeneration. The human miRIDIAN miRNA Mimic Library v.21.0 (384-well format, Dharmacon) was used as a source of miRNA mimics. The miRNA mimic stock solution (1 μM) was dispensed in the test plate (384 well, black with optically clear bottom, PerkinElmer) using the STAR-let liquid handling system (Hamilton). Each well was loaded with a different miRNA-mimic. 661W cells in 384-well plates were transfected with 25 nM of miRNA mimic using a reverse transfection protocol. Briefly, 661W cells (~650 cells/well in Dulbecco's modified Eagle's medium [DMEM]) were loaded using the E1-ClipTip- Electronic Multichannel Pipette (Thermo Fisher Scientific). Pilot experiments were carried out to calculate transfection efficiency using a non-targeting miRNA mimic labeled with Dy547 (Dharmacon). Through such assays, the average transfection efficiency was verified to be approximately 50%. After transfection, the cells were left to recover in the cell culture laminar flow for 5–10 min and then incubated in optimal conditions (5% CO_2, 37°C). About 63 h later, the cells were exposed to white LED light (~15,000 lux) for 6 h. Based on recent studies,[25,26,60] we did not pretreat the cells with a 9-*cis* retinal. Following light exposure, Hoechst 33342 dye was added to the media and incubated for 15 min, and finally the cells were imaged using the Opera/Operetta live imaging system (PerkinElmer). The Columbus software was used for the automated analysis of images. The experiment was performed in three replicates per plate. For the calculation of Z scores from the percentages of dying cells, the following Z score formula was used: $Z = (x - \mu)/\sigma$, where x is the percentage of dying cells in the sample well, μ is the percentage of dying cells in the control wells exposed to light, and σ is the standard deviation of the percentage of dying cells in control wells exposed to light.[61] The selection of cell-protective miRNAs was based on the evaluation of Z scores, CV values, and presence in MirGeneDB.

Cell culture and transfection

661W cells were kindly provided by Dr. Muayyad R. Al-Ubaidi.[19,20] 661W cells were maintained in high-glucose (4.5 mg/mL) DMEM (Life Technologies) containing 10% heat-inactivated fetal bovine serum (FBS; Euroclone), 2 mM L-glutamine, 100 U/mL penicillin, and 100 μg (μg)/mL streptomycin. Cells were never exposed to direct light during handling and were cultured in an incubator with a humidified atmosphere, 5% CO_2, and 37°C. For light-exposure experiments, the medium was replaced by serum-free high-glucose DMEM just before light exposure to sensitize cells to light-induced damage, as previously reported.[21] All reverse transfections in 661W cells were performed with INTERFERin transfection reagent (Polyplus) mixed with Opti-MEM reduced serum media (Thermo Fisher Scientific) following the manufacturer's instructions.

Trypan blue viability assay

661W cells were transfected with 25 nM of miRNA mimics through a reverse transfection protocol in 12-well plates. About 60,000 cells/well were used for transfection. In control wells, the cells were transfected with a negative miRNA mimic. After transfection, the plates were left for 10 min in the cell culture laminar flow to recover and then incubated in optimal conditions for about 45 h. The plates were then exposed to white LED light (~15,000 lux) for 3 h (light condition), harvested immediately, washed with phosphate-buffered saline (PBS), and stained with trypan blue dye. The percentage of dead cells was calculated for each sample by manual cell counting with a Bürker chamber. In parallel, untreated control cells (dark condition) were transfected and incubated

together with the treated cells but in a different 12-well plate, which was covered to prevent any light exposure of the cells. Each of the six miRNA mimics tested was analyzed in duplicate per condition. Three biological replicates were performed for each experiment.

Plasmid construction and AAV production

Recombinant AAV vectors containing the human precursor sequence of hsa-miR-429 under the CMV promoter were constructed by a two-step cloning protocol. Initially, the cassette containing the miR-429 precursor was amplified from human genomic DNA using the following oligonucleotides, 5′-GCGGCCGCCTGTACCCCCACACAGCCAG-3′ and 5′-GGATCCCACCAGGCCATGGCGAGGGTG-3′, and subcloned in a pAAV2.1-CMV-EGFP plasmid[32] from which the EGFP cassette had been previously removed. Recombinant AAV8 viruses (AAV.CMV.miR-429) were produced by the Telethon Institute of Genetics and Medicine (TIGEM) Vector Core as reported.[62,63]

Animal models and procedures

All studies on mice were performed in accordance with the institutional guidelines for animal research (ARRIVE guidelines) and were approved by the Italian Ministry of Health, Department of Public Health, Animal Health, Nutrition and Food Safety, in accordance with the law on animal experimentation (article 7; D.L. 116/92; protocol no. 254/2018-PR). All treatments were approved in advance by the TIGEM Institutional Ethics Committee. Mice were maintained under specific pathogen-free (SPF)-like conditions at the TIGEM Animal Facility. The animals were periodically examined to confirm they were not contaminated with pathogens or infectious diseases. Maintenance of the mice was on a regular 12/12 h light/dark cycle, with a temperature of 20°C–24°C, humidity of 54%–65%, and *ad libitum* access to food and water. For subretinal injections in the $Rho^{P23H/+}$ mouse model, pups were obtained by crossing $Rho^{P23H/P23H}$ knockin mice[34] with C57BL/6J (JAX mice strain, Charles Rivers Laboratories, strain code 632). The C57BL/6J strain was used as a WT control line. $Rho^{P23H/+}$ genotyping was performed with the primer sequences GenoRhoL 2163, 5′-TGGAAGGTCAATGAGGCTCT-3′, and GenoRhoR 2561, 5′-GACCCCACAGAGACAAGCTC-3′, as previously described.[34]

Surgical procedures were performed under anesthesia, and every effort was made to minimize suffering. Viral vectors were delivered subretinally in the temporal retinal areas via a transscleral, transchoroidal approach.[64] Eyes were injected with 1 μL of AAV containing a total of about 1×10^{13} viral genome copies (GCs). Control eyes were injected with an AAV.CMV.EGFP virus, while the AAV.CMV.miR-429 injection consisted of a mixture of AAV.CMV.miR-429 (9/10 of total amount) and AAV.CMV.EGFP (1/10 of total amount) viruses. The latter co-injection served the scope of indirectly monitoring AAV.CMV.miR-429 distribution in injected retinas.

ERG

Scotopic and photopic electrophysiological recordings were performed as previously reported.[65] Dark-adapted animals were anesthetized, their body temperature was maintained at 37.5°C, and the ERG test was carried out according to the formerly described procedure.[16]

Immunofluorescence analysis

Prior to enucleation, each eyeball was orientated by cauterizing the sclerae on their nasal area. Immediately after enucleation, the eyeballs were fixed overnight in 4% PFA, cryoprotected with 30% sucrose, embedded in optimal cutting temperature compound (OCT; Kaltek Srl), and cryosectioned. Cryosections of 12–14 μm were collected on slides (Superfrost Plus; Fisher Scientific, Pittsburgh, PA). For microglia staining, permeabilization and blocking of sections for anti-IBA1 (1:300; 019-19741; Fujifilm Wako Pure Chemical, Osaka, Japan) and anti-MHC class II (1:200; MCA46GA; Bio-Rad, Hercules, CA, USA) primary antibodies were performed for 1 h in 0.3% Triton/4% normal goat serum. Primary antibodies were incubated at 4°C in 0.1% Triton/2% normal goat serum overnight. For rhodopsin and C-arrestin immunostaining, permeabilization of the section was obtained by incubation with 1% NP40 for 15 min. Anti-rhodopsin (Abcam, ab3267, 1:5,000) and anti-C-arrestin (Millipore, 1:1,000) primary antibodies were incubated overnight at 4°C. Sections were then incubated with the secondary antibodies for 2 h (Alexa Fluor 594, anti-rabbit or anti-mouse, 1:1,000, Invitrogen; Alexa Fluor 647, anti-mouse, 1:1,000, Invitrogen) and counterstained with DAPI (Vector Laboratories). All immunofluorescence staining images were acquired using a Zeiss LSM700 confocal microscope. To quantify microglia activation, two pictures from each section were taken in comparable regions of the retina. The analysis was carried out on $N \geq 3$ eyes/treatment. Only retinal sections that contained the optic nerve were analyzed. The ImageJ software was used to convert images to grayscale and normalize background staining. The mean intensity of MHC class II staining was measured at the selected image areas including only ONLs with subretinal space. Finally, the mean intensities per area were calculated.

RNA extraction

Eye samples were processed in QIAzol Lysis Reagent (QIAGEN). RNA was extracted using the miRNeasy extraction kit (QIAGEN) according to the manufacturer's instructions. For the detection of miR-429, RNA was extracted from whole-eye tissue, while for RNA-seq analysis, the RNA was extracted from optic cups.

Reverse transcription and real-time qPCR

Quantitative real-time PCR (real-time qPCR) assays were performed using the miRCURY LNA RT kit (Qiagen) for reverse transcription and miRCURY LNA SYBR Green (Qiagen) for miRNA detection. The real-time qPCR results obtained by a Light Cycler 480 instrument (Roche) and recorded as threshold cycle numbers (Ct) were normalized to the miR-191. miR-191 has been reported to be expressed in mammalian retinas[41,66] and was used as a reference. The real-time qPCR results are presented as $\Delta\Delta$Ct values indicating the ΔCt difference of miR-429 from the ΔCt of the reference. Lower $\Delta\Delta$Ct values indicate higher expression of miR-429, and vice versa.

RNA-seq: Sample preparation and data analysis

Total RNA from optic-cups of $Rho^{P23H/+}$ mice was quantified using a NanoDrop ND-8000 spectrophotometer (NanoDrop Technologies), and the integrity was evaluated using an RNA ScreenTape Assay kit for the Agilent 4200 TapeStation (Agilent Technologies, Santa Clara,

CA, USA). The RNA of the 14 samples had an average RNA integrity number (RIN) of 8.4 (ranging from 7.9 to 8.8). Libraries were prepared according to the manufacturer's instructions (TruSeq RNA Sample Preparation kit, Illumina, San Diego, CA, USA) with an initial amount of 150 ng of total RNA. Quality control of library templates was performed using a High Sensitivity DNA Assay kit (Agilent Technologies) on a 4200 TapeStation (Agilent Technologies). The Qubit quantification platform was used to normalize samples for the library preparation (Qubit 2.0 Fluorometer, Life Technologies). Libraries were sequenced via a paired-end chemistry on an Illumina NovaSeq 6000 platform with an average yield of ~10.6 Gbp. Raw RNA-seq reads were trimmed for Illumina adapters and filtered for low-quality sequences using TrimGalore v.0.6.7 (http://github.com/FelixKrueger/TrimGalore). FastQC v.0.11.9[67] was used to control the quality of the sequencing data at each pre-processing step. Trimmed sequences were mapped against the GRCm39 reference genome using STAR v.2.7.10a.[68] Gene features were counted using HTSeq-count v.2.0.2[69] with Gencode reference annotation v.M31. Two experimental batches were corrected with ComBat-seq.[70] Differential expression analysis was carried out with DESeq2 v.1.38.3[71] taking p.adj < 0.05 and log fold change (logFC) > 1 as the significance threshold. The fgsea bioconductor package v.1.24.0 (https://doi.org/10.1101/060012) was used for the GSEA. The Gene Ontology Biological Process (GOBP) gene set was obtained from the Molecular Signatures Database (MsigDB). The list of enriched biological processes was reduced using the rrvgo bioconductor package v.1.10.0.[72]

ONL and INL thickness analysis

Mouse eyes injected at P8 were orientated and enucleated at P110 and then fixed in 4% PFA, cryoprotected with 30% sucrose, and embedded in OCT. Cryosections of 12–14 μm were collected on glass slides (Superfrost Plus; Fisher Scientific), washed three times with PTW1×, and incubated with DAPI in PBS 1× for 10 min. Sections containing the optic nerve were photographed (at least two sections per eye) with a Leica DM-5500 microscope. The thicknesses of the ONL and INL were manually assessed using ImageJ software at eight locations per section: four measurements toward the dorsal region (0.5, 1.0, 1.5, and 2 mm away from optic nerve head) and four toward the ventral region (0.5, 1.0, 1.5, and 2 mm away from optic nerve head). The analysis was carried out on $N \geq 3$ eyes/treatment.

OKR

Visual acuity in mice was tested with the Optomotor system (OptoMotry; Cerebral Mechanics) as previously reported.[16,73] Animals were injected at P8 and analyzed at three time points (P40, P60, and P110). P30 was not selected as the initial time point of analysis for this test since previous reports showed that the OKR in this mouse model starts to decline around P40.[74] The maximum spatial frequency perceived by each animal was recorded as a threshold of visual acuity. The results are plotted as cycles/degree on the y axis.

SD-OCT

SD-OCT images were obtained using the Bioptigen Spectral Domain Ophthalmic Imaging System (SDOIS; Envisu R2200, Bioptigen, Morrisville, NC, USA). Mice were anesthetized, and pupils were dilated with 1–2 drops of topical 0.5% tropicamide (Visufarma, Rome, Italy). Throughout the process, topical lubricant eye drops (Recugel; Bausch & Lomb, Rochester, NY, USA) were bilaterally administered with a small brush to prevent corneal desiccation. Mice were placed in the animal imaging mount (AIM-RAS; Bioptigen) with their head in a straight, forward-facing position; the laser source was placed in front of the mouse, and images were acquired by the InVivoVue Clinic software (Bioptigen). The protocol involved a linear scan with a 1.4 mm length, 0° angle, 1,000 lines of A scans/B scans, 20 frames, and 8 repeats. Photographs were captured on the temporal and nasal sides from both eyes. Mice injected at P8 were analyzed at three time points (P30, P60, and P110). ONL thickness was evaluated at a minimum of 3 distinct locations on each retina. Data are represented as the average ONL thickness (μm) from at least 3 eyes per treatment.

Statistical analysis

For the trypan blue viability assay, a two-way ANOVA test was performed for each miRNA. For the *in vivo* analyses, the animals were randomly allocated to experimental and control groups based on the appropriate genotype/conditions/treatments. All experiments were carried out blinded. The exact number of experimental replicates is indicated in the legend of each figure. For ERG analyses and ONL/INL thickness spider graphs, the significance of differences between groups of animals was evaluated by two-way ANOVA or a mixed-effects model (in case of missing values). To quantify microglia staining, an unpaired Welch's t test was used. For OKR and SD-OCT assessments, either a t test (paired) or Wilcoxon test was applied. Multiple comparisons were accounted for with Sidak's test. p.adj $\leq$ 0.05 were considered statistically significant. Data are displayed as the mean ± SEM (standard error of the mean) of at least three independent experiments.

DATA AND CODE AVAILABILITY

All data needed to evaluate the conclusions in the paper are present in the paper. The raw sequencing files for the RNA-seq experiments are publicly available on Gene Expression Omnibus (GEO: GSE272674).

ACKNOWLEDGMENTS

We are grateful to Dr. Muayyad R. Al-Ubaidi (University of Oklahoma) for providing us with the 661W cell line. We thank Drs. Brunella Franco, Alberto Auricchio, Enrico Maria Surace, and Cathal Wilson for the critical reading of the manuscript. We also thank Edoardo Nusco, Eugenio Del Prete, and the Retinal Phenotyping, High Content Screening, and Bioinformatics TIGEM Facilities for technical support. The graphical abstract was generated with the use of BioRender (www.biorender.com). This work was supported by the Italian Telethon Foundation (to S.B.); the European Union under the Next Generation EU program, project no. PE00000006 CUP H93C22000660006-MNESYS (to S.B.); and the PON Green/Innovation 2014–2020, D.M. 1062/2021, Action IV.4 "Innovation" (to M.K.).

AUTHOR CONTRIBUTIONS

S.B. conceived the study and designed the experiments; G.P., I.G., S.C., and S.B. wrote the manuscript; G.P., I.G., M.D.G., E.M., M.P., A.T., M.Z., and M.K. carried out the experimental work; S.N. analyzed the transcriptomic data; D.L.M. provided critical expertise for high-content screening assays; and S.B. provided funding for this study. All authors discussed the results and had the opportunity to comment on the manuscript

DECLARATION OF INTERESTS

The authors declare no competing interests.

SUPPLEMENTAL INFORMATION

Supplemental information can be found online at https://doi.org/10.1016/j.omtn.2024.102434.

REFERENCES

1. Hoon, M., Okawa, H., Della Santina, L., and Wong, R.O.L. (2014). Functional architecture of the retina: development and disease. Prog. Retin. Eye Res. *42*, 44–84. https://doi.org/10.1016/j.preteyeres.2014.06.003.
2. Wright, A.F., Chakarova, C.F., Abd El-Aziz, M.M., and Bhattacharya, S.S. (2010). Photoreceptor degeneration: genetic and mechanistic dissection of a complex trait. Nat. Rev. Genet. *11*, 273–284. https://doi.org/10.1038/nrg2717.
3. Newton, F., and Megaw, R. (2020). Mechanisms of Photoreceptor Death in Retinitis Pigmentosa. Genes *11*, 1120. https://doi.org/10.3390/genes11101120.
4. Michaelides, M., Hardcastle, A.J., Hunt, D.M., and Moore, A.T. (2006). Progressive cone and cone-rod dystrophies: phenotypes and underlying molecular genetic basis. Surv. Ophthalmol. *51*, 232–258. https://doi.org/10.1016/j.survophthal.2006.02.007.
5. Bighinati, A., Adani, E., Stanzani, A., D'Alessandro, S., and Marigo, V. (2024). Molecular mechanisms underlying inherited photoreceptor degeneration as targets for therapeutic intervention. Front. Cell. Neurosci. *18*, 1343544. https://doi.org/10.3389/fncel.2024.1343544.
6. Berger, W., Kloeckener-Gruissem, B., and Neidhardt, J. (2010). The molecular basis of human retinal and vitreoretinal diseases. Prog. Retin. Eye Res. *29*, 335–375. https://doi.org/10.1016/j.preteyeres.2010.03.004.
7. Cremers, F.P.M., Boon, C.J.F., Bujakowska, K., and Zeitz, C. (2018). Special Issue Introduction: Inherited Retinal Disease: Novel Candidate Genes, Genotype–Phenotype Correlations, and Inheritance Models. Genes *9*, 215. https://doi.org/10.3390/genes9040215.
8. Ledford, H. (2017). FDA advisers back gene therapy for rare form of blindness. Nature *550*, 314. https://doi.org/10.1038/nature.2017.22819.
9. Brown, E.E., DeWeerd, A.J., Ildefonso, C.J., Lewin, A.S., and Ash, J.D. (2019). Mitochondrial oxidative stress in the retinal pigment epithelium (RPE) led to metabolic dysfunction in both the RPE and retinal photoreceptors. Redox Biol. *24*, 101201. https://doi.org/10.1016/j.redox.2019.101201.
10. Zhao, L., Zabel, M.K., Wang, X., Ma, W., Shah, P., Fariss, R.N., Qian, H., Parkhurst, C.N., Gan, W.B., and Wong, W.T. (2015). Microglial phagocytosis of living photoreceptors contributes to inherited retinal degeneration. EMBO Mol. Med. *7*, 1179–1197. https://doi.org/10.15252/emmm.201505298.
11. Carrella, S., Indrieri, A., Franco, B., and Banfi, S. (2020). Mutation-Independent Therapies for Retinal Diseases: Focus on Gene-Based Approaches. Front. Neurosci. *14*, 588234. https://doi.org/10.3389/fnins.2020.588234.
12. Karali, M., and Banfi, S. (2019). Non-coding RNAs in retinal development and function. Hum. Genet. *138*, 957–971. https://doi.org/10.1007/s00439-018-1931-y.
13. Zuzic, M., Rojo Arias, J.E., Wohl, S.G., and Busskamp, V. (2019). Retinal miRNA Functions in Health and Disease. Genes *10*, 377. https://doi.org/10.3390/genes10050377.
14. Pawlick, J.S., Zuzic, M., Pasquini, G., Swiersy, A., and Busskamp, V. (2020). MiRNA Regulatory Functions in Photoreceptors. Front. Cell Dev. Biol. *8*, 620249. https://doi.org/10.3389/fcell.2020.620249.
15. Conte, I., Hadfield, K.D., Barbato, S., Carrella, S., Pizzo, M., Bhat, R.S., Carissimo, A., Karali, M., Porter, L.F., Urquhart, J., et al. (2015). MiR-204 is responsible for inherited retinal dystrophy associated with ocular coloboma. Proc. Natl. Acad. Sci. USA *112*, E3236–E3245. https://doi.org/10.1073/pnas.1401464112.
16. Carrella, S., Di Guida, M., Brillante, S., Piccolo, D., Ciampi, L., Guadagnino, I., Garcia Piqueras, J., Pizzo, M., Marrocco, E., Molinari, M., et al. (2022). miR-181a/b downregulation: a mutation-independent therapeutic approach for inherited retinal diseases. EMBO Mol. Med. *14*, e15941. https://doi.org/10.15252/emmm.202215941.
17. Karali, M., Guadagnino, I., Marrocco, E., De Cegli, R., Carissimo, A., Pizzo, M., Casarosa, S., Conte, I., Surace, E.M., and Banfi, S. (2020). AAV-miR-204 Protects from Retinal Degeneration by Attenuation of Microglia Activation and Photoreceptor Cell Death. Mol. Ther. Nucleic Acids *19*, 144–156. https://doi.org/10.1016/j.omtn.2019.11.005.
18. Anasagasti, A., Lara-López, A., Milla-Navarro, S., Escudero-Arrarás, L., Rodríguez-Hidalgo, M., Zabaleta, N., González Aseguinolaza, G., de la Villa, P., and Ruiz-Ederra, J. (2020). Inhibition of MicroRNA 6937 Delays Photoreceptor and Vision Loss in a Mouse Model of Retinitis Pigmentosa. Pharmaceutics *12*, 913. https://doi.org/10.3390/pharmaceutics12100913.
19. al-Ubaidi, M.R., Font, R.L., Quiambao, A.B., Keener, M.J., Liou, G.I., Overbeek, P.A., and Baehr, W. (1992). Bilateral retinal and brain tumors in transgenic mice expressing simian virus 40 large T antigen under control of the human interphotoreceptor retinoid-binding protein promoter. J. Cell Biol. *119*, 1681–1687. https://doi.org/10.1083/jcb.119.6.1681.
20. Tan, E., Ding, X.-Q., Saadi, A., Agarwal, N., Naash, M.I., and Al-Ubaidi, M.R. (2004). Expression of Cone-Photoreceptor–Specific Antigens in a Cell Line Derived from Retinal Tumors in Transgenic Mice. Invest. Ophthalmol. Vis. Sci. *45*, 764–768. https://doi.org/10.1167/iovs.03-1114.
21. Kanan, Y., Moiseyev, G., Agarwal, N., Ma, J.-X., and Al-Ubaidi, M.R. (2007). Light induces programmed cell death by activating multiple independent proteases in a cone photoreceptor cell line. Invest. Ophthalmol. Vis. Sci. *48*, 40–51. https://doi.org/10.1167/iovs.06-0592.
22. Natoli, R., Jiao, H., Barnett, N.L., Fernando, N., Valter, K., Provis, J.M., and Rutar, M. (2016). A model of progressive photo-oxidative degeneration and inflammation in the pigmented C57BL/6J mouse retina. Exp. Eye Res. *147*, 114–127. https://doi.org/10.1016/j.exer.2016.04.015.
23. Wenzel, A., Grimm, C., Samardzija, M., and Remé, C.E. (2005). Molecular mechanisms of light-induced photoreceptor apoptosis and neuroprotection for retinal degeneration. Prog. Retin. Eye Res. *24*, 275–306. https://doi.org/10.1016/j.preteyeres.2004.08.002.
24. Chang, Q., Berdyshev, E., Cao, D., Bogaard, J.D., White, J.J., Chen, S., Shah, R., Mu, W., Grantner, R., Bettis, S., and Grassi, M.A. (2014). Cytochrome P450 2C epoxygenases mediate photochemical stress-induced death of photoreceptors. J. Biol. Chem. *289*, 8337–8352. https://doi.org/10.1074/jbc.M113.507152.
25. Li, H., Lian, L., Liu, B., Chen, Y., Yang, J., Jian, S., Zhou, J., Xu, Y., Ma, X., Qu, J., and Hou, L. (2020). KIT ligand protects against both light-induced and genetic photoreceptor degeneration. Elife *9*, e51698. https://doi.org/10.7554/eLife.51698.
26. Natoli, R., Rutar, M., Lu, Y.-Z., Chu-Tan, J.A., Chen, Y., Saxena, K., Madigan, M., Valter, K., and Provis, J.M. (2016). The Role of Pyruvate in Protecting 661W Photoreceptor-Like Cells Against Light-Induced Cell Death. Curr. Eye Res. *41*, 1473–1481. https://doi.org/10.3109/02713683.2016.1139725.
27. Helmy, I.M., and Azim, A.M.A. (2012). Efficacy of ImageJ in the assessment of apoptosis. Diagn. Pathol. *7*, 15. https://doi.org/10.1186/1746-1596-7-15.
28. Eidet, J.R., Pasovic, L., Maria, R., Jackson, C.J., and Utheim, T.P. (2014). Objective assessment of changes in nuclear morphology and cell distribution following induction of apoptosis. Diagn. Pathol. *9*, 92. https://doi.org/10.1186/1746-1596-9-92.
29. Crowley, L.C., Marfell, B.J., and Waterhouse, N.J. (2016). Analyzing Cell Death by Nuclear Staining with Hoechst 33342. Cold Spring Harb. Protoc. *2016*, pdb.prot087205. https://doi.org/10.1101/pdb.prot087205.
30. Fromm, B., Domanska, D., Høye, E., Ovchinnikov, V., Kang, W., Aparicio-Puerta, E., Johansen, M., Flatmark, K., Mathelier, A., Hovig, E., et al. (2020). MirGeneDB 2.0: the metazoan microRNA complement. Nucleic Acids Res. *48*, D132–D141. https://doi.org/10.1093/nar/gkz885.
31. Fromm, B., Billipp, T., Peck, L.E., Johansen, M., Tarver, J.E., King, B.L., Newcomb, J.M., Sempere, L.F., Flatmark, K., Hovig, E., and Peterson, K.J. (2015). A Uniform System for the Annotation of Vertebrate microRNA Genes and the Evolution of the Human microRNAome. Annu. Rev. Genet. *49*, 213–242. https://doi.org/10.1146/annurev-genet-120213-092023.
32. Allocca, M., Mussolino, C., Garcia-Hoyos, M., Sanges, D., Iodice, C., Petrillo, M., Vandenberghe, L.H., Wilson, J.M., Marigo, V., Surace, E.M., and Auricchio, A. (2007). Novel adeno-associated virus serotypes efficiently transduce murine photoreceptors. J. Virol. *81*, 11372–11380. https://doi.org/10.1128/JVI.01327-07.
33. Sakami, S., Kolesnikov, A.V., Kefalov, V.J., and Palczewski, K. (2014). P23H opsin knock-in mice reveal a novel step in retinal rod disc morphogenesis. Hum. Mol. Genet. *23*, 1723–1741. https://doi.org/10.1093/hmg/ddt561.

34. Sakami, S., Maeda, T., Bereta, G., Okano, K., Golczak, M., Sumaroka, A., Roman, A.J., Cideciyan, A.V., Jacobson, S.G., and Palczewski, K. (2011). Probing mechanisms of photoreceptor degeneration in a new mouse model of the common form of autosomal dominant retinitis pigmentosa due to P23H opsin mutations. J. Biol. Chem. *286*, 10551–10567. https://doi.org/10.1074/jbc.M110.209759.

35. Lee, E.-J., Chan, P., Chea, L., Kim, K., Kaufman, R.J., and Lin, J.H. (2021). ATF6 is required for efficient rhodopsin clearance and retinal homeostasis in the P23H rho retinitis pigmentosa mouse model. Sci. Rep. *11*, 16356. https://doi.org/10.1038/s41598-021-95895-7.

36. Comitato, A., Schiroli, D., La Marca, C., and Marigo, V. (2019). Differential Contribution of Calcium-Activated Proteases and ER-Stress in Three Mouse Models of Retinitis Pigmentosa Expressing P23H Mutant RHO. Adv. Exp. Med. Biol. *1185*, 311–316. https://doi.org/10.1007/978-3-030-27378-1_51.

37. Bartel, D.P., and Chen, C.-Z. (2004). Micromanagers of gene expression: the potentially widespread influence of metazoan microRNAs. Nat. Rev. Genet. *5*, 396–400. https://doi.org/10.1038/nrg1328.

38. Fan, W., Huang, W., Chen, J., Li, N., Mao, L., and Hou, S. (2022). Retinal microglia: Functions and diseases. Immunology *166*, 268–286. https://doi.org/10.1111/imm.13479.

39. Noailles, A., Fernández-Sánchez, L., Lax, P., and Cuenca, N. (2014). Microglia activation in a model of retinal degeneration and TUDCA neuroprotective effects. J. Neuroinflammation *11*, 186. https://doi.org/10.1186/s12974-014-0186-3.

40. Wang, T., Yao, J., Jia, L., Fort, P.E., and Zacks, D.N. (2021). Loss of αA or αB-Crystallin Accelerates Photoreceptor Cell Death in a Mouse Model of P23H Autosomal Dominant Retinitis Pigmentosa. Int. J. Mol. Sci. *23*, 70. https://doi.org/10.3390/ijms23010070.

41. Karali, M., Persico, M., Mutarelli, M., Carissimo, A., Pizzo, M., Singh Marwah, V., Ambrosio, C., Pinelli, M., Carrella, D., Ferrari, S., et al. (2016). High-resolution analysis of the human retina miRNome reveals isomiR variations and novel microRNAs. Nucleic Acids Res. *44*, 1525–1540. https://doi.org/10.1093/nar/gkw039.

42. Krol, J., Loedige, I., and Filipowicz, W. (2010). The widespread regulation of microRNA biogenesis, function and decay. Nat. Rev. Genet. *11*, 597–610. https://doi.org/10.1038/nrg2843.

43. Lumayag, S., Haldin, C.E., Corbett, N.J., Wahlin, K.J., Cowan, C., Turturro, S., Larsen, P.E., Kovacs, B., Witmer, P.D., Valle, D., et al. (2013). Inactivation of the microRNA *-183/96/182* cluster results in syndromic retinal degeneration. Proc. Natl. Acad. Sci. USA *110*, E507–E516. https://doi.org/10.1073/pnas.1212655110.

44. Pawlick, J.S., Zuzic, M., Pasquini, G., Swiersy, A., and Busskamp, V. (2020). MiRNA Regulatory Functions in Photoreceptors. Front. Cell Dev. Biol. *8*, 620249. https://doi.org/10.3389/fcell.2020.620249.

45. Kuse, Y., Ogawa, K., Tsuruma, K., Shimazawa, M., and Hara, H. (2014). Damage of photoreceptor-derived cells in culture induced by light emitting diode-derived blue light. Sci. Rep. *4*, 5223. https://doi.org/10.1038/srep05223.

46. Song, J.-Y., Fan, B., Che, L., Pan, Y.-R., Zhang, S.-M., Wang, Y., Bunik, V., and Li, G.-Y. (2020). Suppressing endoplasmic reticulum stress-related autophagy attenuates retinal light injury. Aging *12*, 16579–16596. https://doi.org/10.18632/aging.103846.

47. Senfter, D., Madlener, S., Krupitza, G., and Mader, R.M. (2016). The microRNA-200 family: still much to discover. Biomol. Concepts *7*, 311–319. https://doi.org/10.1515/bmc-2016-0020.

48. Wheeler, B.M., Heimberg, A.M., Moy, V.N., Sperling, E.A., Holstein, T.W., Heber, S., and Peterson, K.J. (2009). The deep evolution of metazoan microRNAs. Evol. Dev. *11*, 50–68. https://doi.org/10.1111/j.1525-142X.2008.00302.x.

49. Trümbach, D., and Prakash, N. (2015). The conserved miR-8/miR-200 microRNA family and their role in invertebrate and vertebrate neurogenesis. Cell Tissue Res. *359*, 161–177. https://doi.org/10.1007/s00441-014-1911-z.

50. Klicka, K., Grzywa, T.M., Mielniczuk, A., Klinke, A., and Włodarski, P.K. (2022). The role of miR-200 family in the regulation of hallmarks of cancer. Front. Oncol. *12*, 965231. https://doi.org/10.3389/fonc.2022.965231.

51. Xu, H., Yang, B., Ren, Z., Wu, D., Hu, A., and Hu, J. (2022). miR-429 negatively regulates the progression of hypoxia-induced retinal neovascularization by the HPSE-VEGF pathway. Exp. Eye Res. *223*, 109196. https://doi.org/10.1016/j.exer.2022.109196.

52. Vasudevan, S., Senapati, S., Pendergast, M., and Park, P.S.–H. (2024). Aggregation of rhodopsin mutants in mouse models of autosomal dominant retinitis pigmentosa. Nat. Commun. *15*, 1451. https://doi.org/10.1038/s41467-024-45748-4.

53. Chiang, W.-C., Kroeger, H., Sakami, S., Messah, C., Yasumura, D., Matthes, M.T., Coppinger, J.A., Palczewski, K., LaVail, M.M., and Lin, J.H. (2015). Robust Endoplasmic Reticulum-Associated Degradation of Rhodopsin Precedes Retinal Degeneration. Mol. Neurobiol. *52*, 679–695. https://doi.org/10.1007/s12035-014-8881-8.

54. Natkunarajah, M., Trittibach, P., McIntosh, J., Duran, Y., Barker, S.E., Smith, A.J., Nathwani, A.C., and Ali, R.R. (2008). Assessment of ocular transduction using single-stranded and self-complementary recombinant adeno-associated virus serotype 2/8. Gene Ther. *15*, 463–467. https://doi.org/10.1038/sj.gt.3303074.

55. Auricchio, A., Kobinger, G., Anand, V., Hildinger, M., O'Connor, E., Maguire, A.M., Wilson, J.M., and Bennett, J. (2001). Exchange of surface proteins impacts on viral vector cellular specificity and transduction characteristics: the retina as a model. Hum. Mol. Genet. *10*, 3075–3081. https://doi.org/10.1093/hmg/10.26.3075.

56. Wooff, Y., Cioanca, A.V., Chu-Tan, J.A., Aggio-Bruce, R., Schumann, U., and Natoli, R. (2020). Small-Medium Extracellular Vesicles and Their miRNA Cargo in Retinal Health and Degeneration: Mediators of Homeostasis, and Vehicles for Targeted Gene Therapy. Front. Cell. Neurosci. *14*, 160. https://doi.org/10.3389/fncel.2020.00160.

57. Cioanca, A.V., Wooff, Y., Aggio-Bruce, R., Sekar, R., Dietrich, C., and Natoli, R. (2023). Multiomic integration reveals neuronal-extracellular vesicle coordination of gliotic responses in degeneration. J. Extracell. Vesicles *12*, e12393. https://doi.org/10.1002/jev2.12393.

58. Costa, B.L.d., Quinn, P.M.J., Wu, W.-H., Liu, S., Nolan, N.D., Demirkol, A., Tsai, Y.-T., Caruso, S.M., Cabral, T., Wang, N.-K., and Tsang, S.H. (2024). Targeting miR-181a/b in retinitis pigmentosa: implications for disease progression and therapy. Cell Biosci. *14*, 64. https://doi.org/10.1186/s13578-024-01243-3.

59. Gao, C., Jiang, J., Tan, Y., and Chen, S. (2023). Microglia in neurodegenerative diseases: mechanism and potential therapeutic targets. Signal Transduct. Targeted Ther. *8*, 359. https://doi.org/10.1038/s41392-023-01588-0.

60. Bullock, J., Pagan-Mercado, G., and Becerra, S.P. (2020). Cell-based assays to identify novel retinoprotective agents. MethodsX *7*, 101026. https://doi.org/10.1016/j.mex.2020.101026.

61. Zhang, J., Chung, T., and Oldenburg, K. (1999). A Simple Statistical Parameter for Use in Evaluation and Validation of High Throughput Screening Assays. J. Biomol. Screen *4*, 67–73. https://doi.org/10.1177/108705719900400206.

62. Doria, M., Ferrara, A., and Auricchio, A. (2013). AAV2/8 vectors purified from culture medium with a simple and rapid protocol transduce murine liver, muscle, and retina efficiently. Hum. Gene Ther. Methods *24*, 392–398. https://doi.org/10.1089/hgtb.2013.155.

63. Hildinger, M., Auricchio, A., Gao, G., Wang, L., Chirmule, N., and Wilson, J.M. (2001). Hybrid vectors based on adeno-associated virus serotypes 2 and 5 for muscle-directed gene transfer. J. Virol. *75*, 6199–6203. https://doi.org/10.1128/JVI.75.13.6199-6203.2001.

64. Liang, F.-Q., Anand, V., Maguire, A.M., and Bennett, J. (2001). Intraocular Delivery of Recombinant Virus. In Vision Research Protocols (Humana Press), pp. 125–139. https://doi.org/10.1385/1-59259-085-3:125.

65. Barbato, S., Marrocco, E., Intartaglia, D., Pizzo, M., Asteriti, S., Naso, F., Falanga, D., Bhat, R.S., Meola, N., Carissimo, A., et al. (2017). MiR-211 is essential for adult cone photoreceptor maintenance and visual function. Sci. Rep. *7*, 17004. https://doi.org/10.1038/s41598-017-17331-z.

66. Xu, S., Witmer, P.D., Lumayag, S., Kovacs, B., and Valle, D. (2007). MicroRNA (miRNA) Transcriptome of Mouse Retina and Identification of a Sensory Organ-specific miRNA Cluster. J. Biol. Chem. *282*, 25053–25066. https://doi.org/10.1074/jbc.M700501200.

67. Trivedi, U.H., CÃ©zard, T., Bridgett, S., Montazam, A., Nichols, J., Blaxter, M., and Gharbi, K. (2014). Quality control of next-generation sequencing data without a reference. Front. Genet. *5*, 111. https://doi.org/10.3389/fgene.2014.00111.

68. Dobin, A., and Gingeras, T.R. (2015). Mapping RNA-seq Reads with STAR. Curr. Protoc. Bioinformatics *51*, 11.14.1–11.14.19. https://doi.org/10.1002/0471250953.bi1114s51.

69. Anders, S., Pyl, P.T., and Huber, W. (2015). HTSeq–a Python framework to work with high-throughput sequencing data. Bioinformatics *31*, 166–169. https://doi.org/10.1093/bioinformatics/btu638.

70. Zhang, Y., Parmigiani, G., and Johnson, W.E. (2020). ComBat-seq: batch effect adjustment for RNA-seq count data. NAR Genom. Bioinform. *2*, lqaa078. https://doi.org/10.1093/nargab/lqaa078.

71. Love, M.I., Huber, W., and Anders, S. (2014). Moderated estimation of fold change and dispersion for RNA-seq data with DESeq2. Genome Biol. *15*, 550. https://doi.org/10.1186/s13059-014-0550-8.

72. Sayols, S. (2023). rrvgo: a Bioconductor package for interpreting lists of Gene Ontology terms. MicroPubl. Biol. *2023*, 000811. https://doi.org/10.17912/micropub.biology.000811.

73. Indrieri, A., Carrella, S., Romano, A., Spaziano, A., Marrocco, E., Fernandez-Vizarra, E., Barbato, S., Pizzo, M., Ezhova, Y., Golia, F.M., et al. (2019). miR-181a/b downregulation exerts a protective action on mitochondrial disease models. EMBO Mol. Med. *11*, e8734. https://doi.org/10.15252/emmm.201708734.

74. Liu, X., Chen, F., Chen, Y., Lu, H., Lu, X., Peng, X., Kaplan, H.J., Dean, D.C., Gao, L., and Liu, Y. (2020). Paracrine effects of intraocularly implanted cells on degenerating retinas in mice. Stem Cell Res. Ther. *11*, 142. https://doi.org/10.1186/s13287-020-01651-5.

OMTN, Volume *36*

Supplemental information

In vitro high-content screening reveals miR-429 as a protective molecule in photoreceptor degeneration

Georgios Petrogiannakis, Irene Guadagnino, Santiago Negueruela, Martina Di Guida, Elena Marrocco, Mariateresa Pizzo, Annalaura Torella, Mariateresa Zanobio, Marianthi Karali, Diego Luis Medina, Sabrina Carrella, and Sandro Banfi

Table S1: HCI results of all tested miRNAs

Table S2: Gene Ontology biological processes enriched in response to miR-429 overexpression

pathway	pval	padj	log2 err	ES	NES	size
GOBP_INTERLEUKIN_10_PRODUCTION	2.57E-07	1.20E-04	0.67	-0.69	-2.21	53
GOBP_REGULATION_OF_LYMPHOCYTE_MEDIATED_IMMUNITY	9.09E-07	2.06E-04	0.66	-0.51	-1.90	157
GOBP_B_CELL_MEDIATED_IMMUNITY	3.61E-06	4.40E-04	0.63	-0.51	-1.88	143
GOBP_REGULATION_OF_PHAGOCYTOSIS	4.43E-06	5.10E-04	0.61	-0.58	-2.01	86
GOBP_NEGATIVE_REGULATION_OF_LEUKOCYTE_MEDIATED_IMMUNITY	1.69E-05	1.60E-03	0.58	-0.63	-2.09	66
GOBP_NEGATIVE_REGULATION_OF_LYMPHOCYTE_MEDIATED_IMMUNITY	2.01E-05	1.86E-03	0.58	-0.63	-2.02	54
GOBP_CELLULAR_RESPONSE_TO_AMYLOID_BETA	2.48E-05	2.03E-03	0.58	-0.68	-2.05	40
GOBP_NEGATIVE_REGULATION_OF_IMMUNE_EFFECTOR_PROCESS	2.59E-05	2.04E-03	0.58	-0.52	-1.86	107
GOBP_RESPONSE_TO_AMYLOID_BETA	2.72E-05	2.10E-03	0.58	-0.66	-2.06	49
GOBP_HUMORAL_IMMUNE_RESPONSE_MEDIATED_BY_CIRCULATING_IMMUNOGLOBULIN	2.97E-05	2.18E-03	0.58	-0.61	-2.01	64
GOBP_MEMBRANE_INVAGINATION	3.48E-05	2.51E-03	0.56	-0.53	-1.84	87
GOBP_REGULATION_OF_T_CELL_MEDIATED_IMMUNITY	3.84E-05	2.71E-03	0.56	-0.56	-1.92	79
GOBP_MACROPHAGE_ACTIVATION	4.31E-05	2.87E-03	0.56	-0.54	-1.86	95
GOBP_POSITIVE_REGULATION_OF_INTERLEUKIN_10_PRODUCTION	4.59E-05	2.91E-03	0.56	-0.68	-2.00	38
GOBP_POSITIVE_REGULATION_OF_PHAGOCYTOSIS	4.72E-05	2.91E-03	0.56	-0.62	-2.03	58
GOBP_NEGATIVE_REGULATION_OF_CELL_KILLING	5.13E-05	3.12E-03	0.56	-0.71	-1.98	30
GOBP_INTERFERON_GAMMA_PRODUCTION	7.57E-05	4.08E-03	0.54	-0.52	-1.82	101
GOBP_REGULATION_OF_CELL_KILLING	8.90E-05	4.68E-03	0.54	-0.53	-1.85	93
GOBP_CELLULAR_EXTRAVASATION	1.00E-04	5.07E-03	0.54	-0.56	-1.87	67
GOBP_COMPLEMENT_ACTIVATION	1.12E-04	5.47E-03	0.54	-0.56	-1.88	69
GOBP_DETECTION_OF_LIGHT_STIMULUS	1.29E-04	6.23E-03	0.52	0.59	1.86	58
GOBP_LENS_DEVELOPMENT_IN_CAMERA_TYPE_EYE	1.54E-04	7.20E-03	0.52	0.54	1.83	80
GOBP_PHOTORECEPTOR_CELL_DEVELOPMENT	2.25E-04	9.20E-03	0.52	0.61	1.88	48
GOBP_POSITIVE_REGULATION_OF_INTERFERON_GAMMA_PRODUCTION	2.76E-04	1.10E-02	0.50	-0.54	-1.82	71
GOBP_EYE_PHOTORECEPTOR_CELL_DEVELOPMENT	3.05E-04	1.18E-02	0.50	0.64	1.86	34
GOBP_REGULATION_OF_T_CELL_MEDIATED_CYTOTOXICITY	3.25E-04	1.25E-02	0.50	-0.65	-1.87	33
GOBP_DETECTION_OF_VISIBLE_LIGHT	4.24E-04	1.56E-02	0.50	0.61	1.87	45
GOBP_POSITIVE_REGULATION_OF_TYROSINE_PHOSPHORYLATION_OF_STAT_PROTEIN	4.38E-04	1.57E-02	0.50	-0.57	-1.85	56
GOBP_TYROSINE_PHOSPHORYLATION_OF_STAT_PROTEIN	5.23E-04	1.77E-02	0.48	-0.54	-1.78	69
GOBP_REGULATION_OF_LEUKOCYTE_MEDIATED_CYTOTOXICITY	5.43E-04	1.82E-02	0.48	-0.52	-1.76	74
GOBP_POSITIVE_REGULATION_OF_CALCIUM_MEDIATED_SIGNALING	5.76E-04	1.89E-02	0.48	-0.67	-1.90	32
GOBP_REGULATION_OF_B_CELL_MEDIATED_IMMUNITY	6.75E-04	2.14E-02	0.48	-0.55	-1.76	55
GOBP_B_CELL_PROLIFERATION	6.97E-04	2.18E-02	0.48	-0.52	-1.78	82
GOBP_FC_GAMMA_RECEPTOR_SIGNALING_PATHWAY	7.41E-04	2.29E-02	0.48	-0.65	-1.81	30
GOBP_EYE_PHOTORECEPTOR_CELL_DIFFERENTIATION	7.62E-04	2.30E-02	0.48	0.57	1.74	44
GOBP_INTERLEUKIN_12_PRODUCTION	7.62E-04	2.30E-02	0.48	-0.54	-1.78	63
GOBP_NEUROINFLAMMATORY_RESPONSE	7.87E-04	2.34E-02	0.48	-0.62	-1.82	38
GOBP_PHOTORECEPTOR_CELL_DIFFERENTIATION	8.10E-04	2.36E-02	0.48	0.54	1.73	59
GOBP_WATER_HOMEOSTASIS	8.35E-04	2.41E-02	0.48	0.54	1.76	61
GOBP_REGULATION_OF_RECEPTOR_SIGNALING_PATHWAY_VIA_STAT	8.68E-04	2.46E-02	0.48	-0.52	-1.77	82

GOBP_NEGATIVE_REGULATION_OF_TUMOR_NECROSIS_FACTOR_SUPERFAMILY_CYTOKINE_PRODUCTION	8.90E-04	2.48E-02	0.48	-0.53	-1.73	60
GOBP_REGULATION_OF_B_CELL_PROLIFERATION	1.02E-03	2.74E-02	0.46	-0.53	-1.74	62
GOBP_REGULATION_OF_MACROPHAGE_ACTIVATION	1.07E-03	2.77E-02	0.46	-0.55	-1.78	57
GOBP_CYTOKINE_PRODUCTION_INVOLVED_IN_INFLAMMATORY_RESPONSE	1.07E-03	2.77E-02	0.46	-0.58	-1.81	49
GOBP_REGULATION_OF_ANTIGEN_PROCESSING_AND_PRESENTATION	1.05E-03	2.77E-02	0.46	-0.78	-1.86	15
GOBP_POSITIVE_REGULATION_OF_ACUTE_INFLAMMATORY_RESPONSE	1.06E-03	2.77E-02	0.46	-0.69	-1.87	25
GOBP_TETRAPYRROLE_BIOSYNTHETIC_PROCESS	1.09E-03	2.78E-02	0.46	0.64	1.80	30
GOBP_NEGATIVE_REGULATION_OF_ADAPTIVE_IMMUNE_RESPONSE	1.23E-03	3.04E-02	0.46	-0.56	-1.81	58
GOBP_SULFATION	1.28E-03	3.07E-02	0.46	0.71	1.86	21
GOBP_NEGATIVE_REGULATION_OF_EXOCYTOSIS	1.28E-03	3.07E-02	0.46	-0.62	-1.78	33
GOBP_REGULATION_OF_NITRIC_OXIDE_METABOLIC_PROCESS	1.35E-03	3.17E-02	0.46	-0.54	-1.73	53
GOBP_DEFENSE_RESPONSE_TO_GRAM_POSITIVE_BACTERIUM	1.42E-03	3.28E-02	0.46	-0.50	-1.68	73
GOBP_DETECTION_OF_EXTERNAL_BIOTIC_STIMULUS	1.43E-03	3.29E-02	0.46	-0.66	-1.80	27
GOBP_LENS_FIBER_CELL_DIFFERENTIATION	1.47E-03	3.35E-02	0.46	0.59	1.72	36
GOBP_REGULATION_OF_MYELOID_LEUKOCYTE_MEDIATED_IMMUNITY	1.66E-03	3.65E-02	0.46	-0.54	-1.75	56
GOBP_PHOTOTRANSDUCTION	1.71E-03	3.70E-02	0.46	0.58	1.75	41
GOBP_REGULATION_OF_WATER_LOSS_VIA_SKIN	1.73E-03	3.71E-02	0.46	0.65	1.80	27
GOBP_GLUTAMATE_RECEPTOR_SIGNALING_PATHWAY	1.83E-03	3.84E-02	0.46	-0.56	-1.77	51
GOBP_NEGATIVE_REGULATION_OF_POSTTRANSCRIPTIONAL_GENE_SILENCING	1.89E-03	3.89E-02	0.46	-0.76	-1.83	15
GOBP_ACTIVATION_OF_JANUS_KINASE_ACTIVITY	1.92E-03	3.94E-02	0.46	-0.76	-1.82	15
GOBP_MODIFIED_AMINO_ACID_TRANSPORT	2.04E-03	4.14E-02	0.43	0.61	1.75	33
GOBP_REGULATION_OF_ACUTE_INFLAMMATORY_RESPONSE	2.05E-03	4.14E-02	0.43	-0.57	-1.77	45
GOBP_PIGMENT_BIOSYNTHETIC_PROCESS	2.21E-03	4.29E-02	0.43	0.50	1.62	62
GOBP_NEGATIVE_REGULATION_OF_PRODUCTION_OF_MOLECULAR_MEDIATOR_OF_IMMUNE_RESPONSE	2.23E-03	4.29E-02	0.43	-0.62	-1.82	37
GOBP_ANTIFUNGAL_INNATE_IMMUNE_RESPONSE	2.28E-03	4.36E-02	0.43	-0.71	-1.78	18
GOBP_MICROGLIAL_CELL_ACTIVATION	2.37E-03	4.47E-02	0.43	-0.59	-1.77	41
GOBP_POSITIVE_REGULATION_OF_NITRIC_OXIDE_METABOLIC_PROCESS	2.65E-03	4.80E-02	0.43	-0.58	-1.72	38
GOBP_PHOTORECEPTOR_CELL_MAINTENANCE	2.71E-03	4.89E-02	0.43	0.58	1.72	40
GOBP_TOLERANCE_INDUCTION	2.76E-03	4.96E-02	0.43	-0.61	-1.71	30

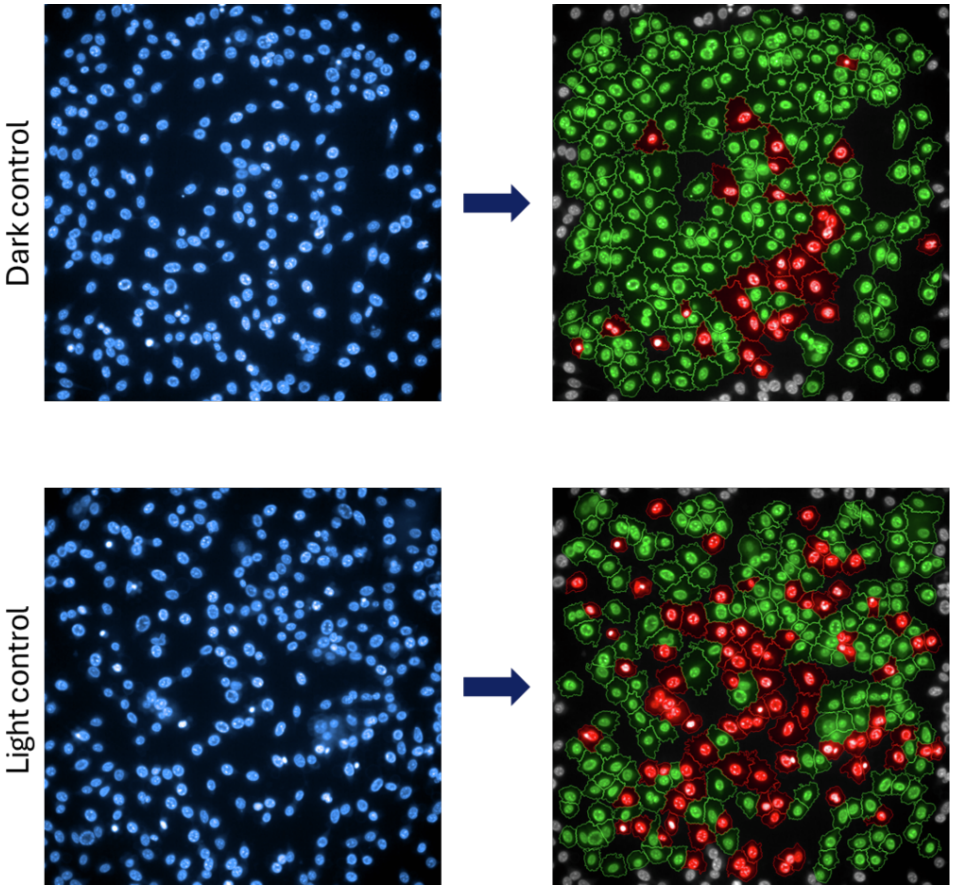

Figure S1: Image automated analysis.

Representative images from Dark and Light controls acquired by the High Content microscope. After Hoechst-dye staining of the nuclei, the automated microscope acquired about 6 pictures per well. The Columbus software detected stained nuclei and characterized each one as 'live' (green colour) or 'dying' (red colour).

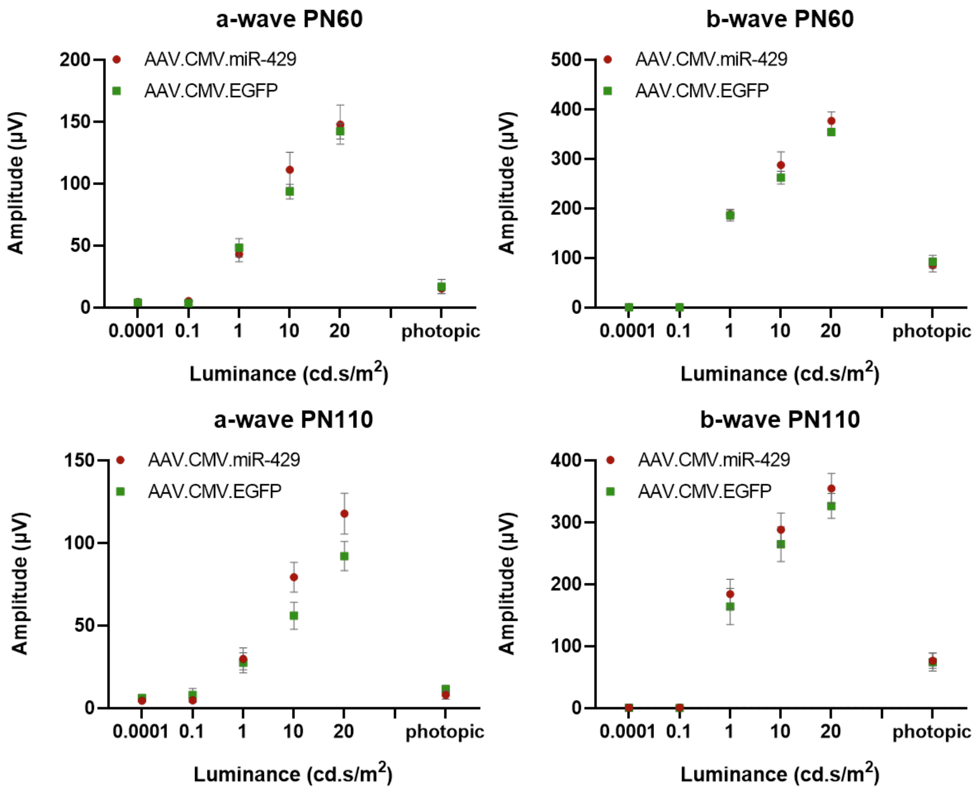

Figure S2: a- and b-wave responses from animals injected at PN30.

ERGs were performed at PN60 (N = 6), and PN110 (N = 7). Data are presented as mean ± SEM.

A.

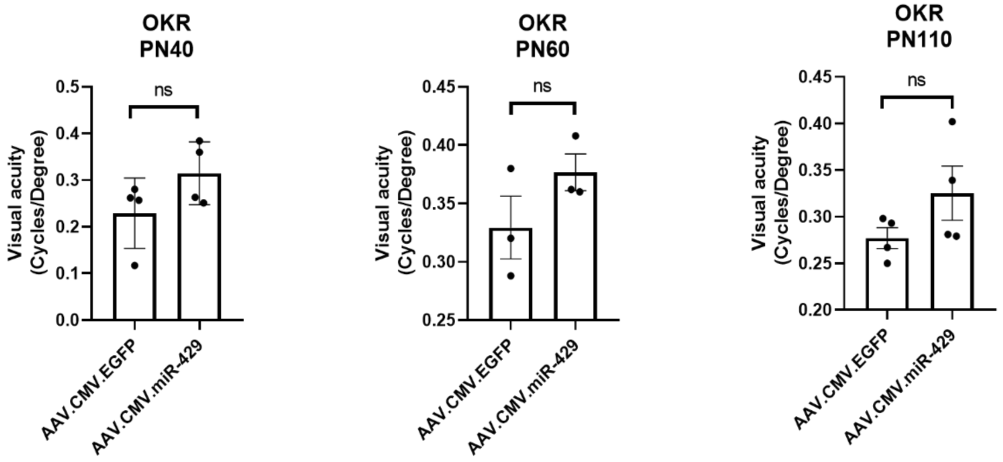

B.

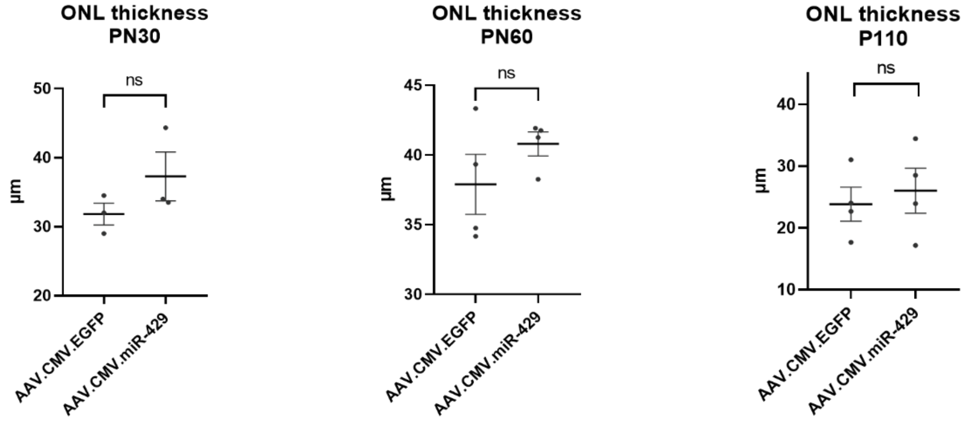

Figure S3: Assessment of visual acuity and retinal morphology in AAV.CMV.miR-429-injected animals.

(A) Optokinetic responses of $Rho^{P23H/+}$ mice injected at PN8 with AAV.CMV.EGFP or AAV.CMV.miR-429 and assayed at PN40 (N = 4), PN60 (N = 3) and PN110 (N = 4). Results are reported as cycles/degree. **(B)** ONL thickness measurements obtained through SD-OCT in $Rho^{P23H/+}$ mice injected at PN8 with AAV.CMV.EGFP or AAV.CMV.miR-429 and assayed at PN30 (N = 3), PN60 (N = 4) and PN110 (N = 4). Data are presented as mean ± SEM. ns: not significant

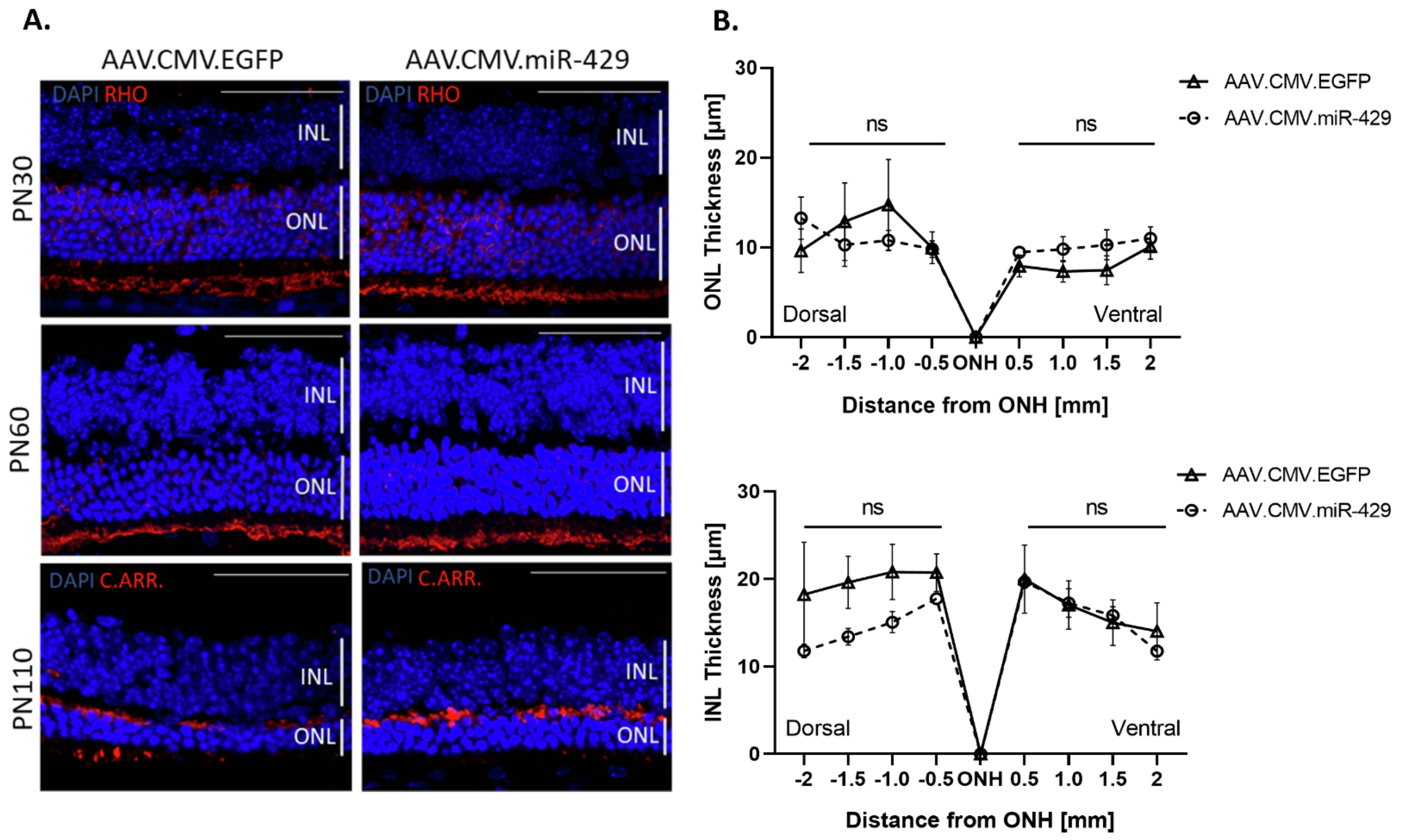

Figure S4: Morphological analysis of AAV.CMV.miR-429-injected eyes.

(A) Immunofluorescence staining for PR markers in $Rho^{P23H/+}$ mice injected at PN8 with AAV.CMV.EGFP or AAV.CMV.miR-429 and assayed at PN30, PN60, and PN110. Rhodopsin (RHO) and Cone Arrestin (C.ARR.) are shown in red. DAPI nuclei counterstaining is shown in blue. INL, inner nuclear layer; ONL, outer nuclear layer. Scale bars: 50 µm. **(B)** Spider graphs showing the ONL and INL thickness of AAV.CMV.miR-429 and AAV.CMV.EGFP-injected retinas at different distances from the optic nerve head (ONH) in the dorsal or ventral retina. AAV.CMV.EGFP. N = 3; AAV.CMV.miR-429. N =4. Data are presented as mean ± SEM. ns: not significant, two-way ANOVA.
